# Dynamic Regulation of H3K27 Trimethylation during *Arabidopsis* Differentiation

**DOI:** 10.1371/journal.pgen.1002040

**Published:** 2011-04-07

**Authors:** Marcel Lafos, Phillip Kroll, Mareike L. Hohenstatt, Frazer L. Thorpe, Oliver Clarenz, Daniel Schubert

**Affiliations:** 1 Institute of Genetics, Heinrich-Heine-University Duesseldorf, Duesseldorf, Germany; 2 Institute for Molecular Plant Sciences, University of Edinburgh, Edinburgh, United Kingdom; National Institute of Genetics, Japan

## Abstract

During growth of multicellular organisms, identities of stem cells and differentiated cells need to be maintained. Cell fate is epigenetically controlled by the conserved Polycomb-group (Pc-G) proteins that repress their target genes by catalyzing histone H3 lysine 27 trimethylation (H3K27me3). Although H3K27me3 is associated with mitotically stable gene repression, a large fraction of H3K27me3 target genes are tissue-specifically activated during differentiation processes. However, in plants it is currently unclear whether H3K27me3 is already present in undifferentiated cells and dynamically regulated to permit tissue-specific gene repression or activation. We used whole-genome tiling arrays to identify the H3K27me3 target genes in undifferentiated cells of the shoot apical meristem and in differentiated leaf cells. Hundreds of genes gain or lose H3K27me3 upon differentiation, demonstrating dynamic regulation of an epigenetic modification in plants. H3K27me3 is correlated with gene repression, and its release preferentially results in tissue-specific gene activation, both during differentiation and in Pc-G mutants. We further reveal meristem- and leaf-specific targeting of individual gene families including known but also likely novel regulators of differentiation and stem cell regulation. Interestingly, H3K27me3 directly represses only specific transcription factor families, but indirectly activates others through H3K27me3-mediated silencing of microRNA genes. Furthermore, H3K27me3 targeting of genes involved in biosynthesis, transport, perception, and signal transduction of the phytohormone auxin demonstrates control of an entire signaling pathway. Based on these and previous analyses, we propose that H3K27me3 is one of the major determinants of tissue-specific expression patterns in plants, which restricts expression of its direct targets and promotes gene expression indirectly by repressing miRNA genes.

## Author Summary

All organs and differentiated tissues in multicellular organisms are derived from undifferentiated pluripotent stem cells. The evolutionarily conserved Polycomb-group (Pc-G) proteins control stem cell identity and maintenance, likely by repressing genes involved in differentiation processes. Pc-G proteins are epigenetic regulators, thus they maintain stable expression states of their target genes through cell divisions that are not accompanied by changes in their DNA sequence. In this study, we asked whether Pc-G–mediated gene regulation is also dynamically regulated in plant development to confer stable, but flexible gene expression states that may switch in response to developmental or environmental cues. We therefore generated genome-wide maps of Pc-G activity of undifferentiated stem cell and differentiated leaf cell tissues which revealed dynamic regulation of Pc-G activity in plants. Pc-G activity is correlated with gene repression and its tissue-specific release results in local gene activation. Pc-G proteins target specific gene families in the two analyzed tissues, indicating a role for Pc-G proteins in balancing pluripotency and differentiation in plants. Based on our analyses, we propose that Pc-G activity not only permits long-term gene regulation but also has a more basic gene regulatory function in fine-tuning expression patterns of specific gene families during differentiation.

## Introduction

Throughout their lifecycle, plants produce new organs through a group of undifferentiated cells which are maintained in structures called meristems. These pluripotent cells continuously divide and, in the shoot, cells in the periphery of the apical meristem differentiate to give rise to lateral organs like leaves or flowers. Stem cell maintenance is tightly controlled by numerous and interconnected pathways involving transcriptional regulation, phytohormones, microRNAs and epigenetic gene regulation (reviewed in [1]).

Epigenetic gene regulation is a key mechanism to confer stable, but reversible gene expression states. Epigenetics has been revealed as fundamental mechanism to maintain cell and tissue identity and to regulate stem cells and cancer.

Important epigenetic regulators of developmental processes are the Polycomb-group (Pc-G) and Trithorax-group (Trx-G) proteins which catalyze histone H3 lysine 27 trimethylation (H3K27me3) or H3K4me3, respectively (reviewed in [2], [3]). In *Arabidopsis*, around 5% of the canonical histone H3.1 is trimethylated at K27 [4]. Pc-G target genes in plants and animals are covered by H3K27me3 [5]–[8], a distribution that likely permits epigenetic inheritance of the modification [9]. Whereas *Drosophila melanogaster* Polycomb repressive complex 2 (PRC2) was shown to methylate H3K27 in vitro and in vivo via its histone methyltransferase subunit Enhancer of zeste [E(z)] [10]–[13], evidence for the biochemical activity of the conserved plant PRC2 is still lacking. H3K27me3 was shown to depend at least partially on plant PRC2 members as loss of the E(z) homolog CURLY LEAF (CLF) leads to reduced levels of H3K27me3 [8], [14]. In addition, immunofluorescence analyses of plants lacking the redundantly acting E(z) homologs CLF and SWINGER (SWN) revealed a reduction in euchromatic H3K27me3, but also a frequent re-distribution to chromocenters [15].

In plants, numerous developmental pathways are controlled by Pc-G proteins including seed development, flowering time, vernalization and organ identity (reviewed in [16], [17]). Loss of sporophytic Pc-G activity results in plants that show overproliferation and strong defects in organ identity [18], [19]. The severe phenotypes of Pc-G mutants indicate an essential role for Pc-G proteins in plant development which was strongly supported by whole genome analyses of H3K27me3 targets. In seedlings, more than 4000 H3K27me3 target genes were uncovered which are largely protein-coding genes and mostly exclude transposable element genes and heterochromatic regions [5], [20], [21]. A strikingly large number of developmentally important transcription factor genes showed H3K27me3 coverage [5]. However, the fact that key genes involved in the biosynthesis and inactivation of the phytohormone gibberellic acid exhibit enrichment in H3K27me3 [22] indicates an important role for Pc-G proteins in regulating developmental processes beyond the transcription factor level. Numerous genome-wide analyses of H3K27me3 and Pc-G protein binding have been performed for mammals and *Drosophila* [6], [23]–[29]. These studies revealed a considerably smaller number of target genes in *Drosophila* and mammals compared to *Arabidopsis*, but also key developmental transcription factor genes as H3K27me3 targets.

Presence of H3K27me3 is largely correlated with gene silencing in animals and plants, although H3K27me3 is only partially removed upon gene activation in *Drosophila* [30]. In *Arabidopsis*, H3K27me3 targets are enriched for genes with tissue-specific expression patterns or are induced by abiotic or biotic stresses suggesting that H3K27me3 is dynamically regulated in response to developmental or environmental cues [5]. Indeed, several thousand genes either lose or gain H3K27me3 when etiolated seedlings were transferred into light [22]. Furthermore, a novel approach of cell-type specific tagging and isolation of nuclei allowing cell type specific analyses of hair and non-hair cells of the *Arabidopsis* root epidermis revealed hundreds of genes carrying differential H3K4me3 and H3K27me3 in the two cell types and a strong correlation of gene repression with low H3K4me3 and high H3K27me3 [31].

Different cell types can harbor distinct chromatin profiles which is particularly the case for pluripotent mammalian embryonic stem (ES) cells [7], [32]. Many genes in ES cells carry bivalent marks, thus both H3K4me3 and H3K27me3, which are mostly resolved to monovalent states of either H3K4me3 or H3K27me3 during differentiation [7], [33]. In plants, however, it is currently unclear if undifferentiated cells harbor distinct chromatin profiles.

Here, we present genome-wide analyses of H3K27me3 as well as gene expression analyses from meristematic, undifferentiated cells and differentiated leaf cells using the vegetative shoot apical meristem as a model system. We confirm a large fraction of previously identified H3K27me3 targets and reveal several hundred additional, tissue-specifically methylated genes. Genes with strong expression differences in the two tissues are enriched for differential H3K27me3 suggesting that also in plants Pc-G proteins define gene ON/OFF expression states. We find that a large fraction of microRNA genes are differentially methylated in meristems and leaves and that their tissue-specific expression patterns are controlled by Pc-G proteins. In addition, a large number of genes involved in biosynthesis, transport, perception and signaling of the phytohormone auxin are among the identified target genes suggesting that entire gene regulatory networks are controlled and possibly stabilized by Pc-G mediated gene regulation. Collectively, our analyses suggest that Pc-G proteins control differentiation processes by conferring tissue-specific H3K27me3 of hundreds of genes including microRNA genes and genes involved hormonal pathways.

## Results

### Genome-wide analyses of gene expression and H3K27me3 in meristems and leaves

In *Arabidopsis* seedlings, several thousand genes are covered by H3K27me3 [5], [20].

**Figure 1:**
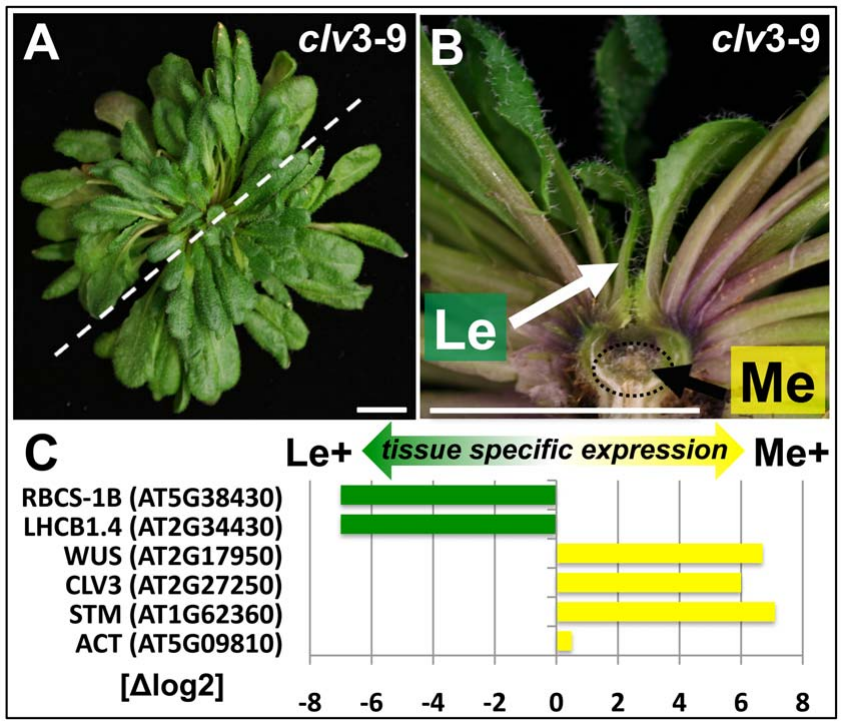
Tissue enrichment through manual dissection of *Arabidopsis clv3* mutants. (A) Nine week old *clv3*–*9* plant grown under short day conditions, (B) longitudinal section of plant shown in (A). The section plane is marked with a dashed line in A, scale bars indicate 1 cm. Two samples, a meristematic fraction (Me) marked with a dotted circle and young leaves (Le) were harvested. (C) Differential expression of known reference genes obtained by genome wide expression analysis in the two dissected tissues. *CLV3* expression is detected as the point mutation in the *clv3*–*9* allele does not interfere with *CLV3* transcriptional regulation.

**Figure 2:**
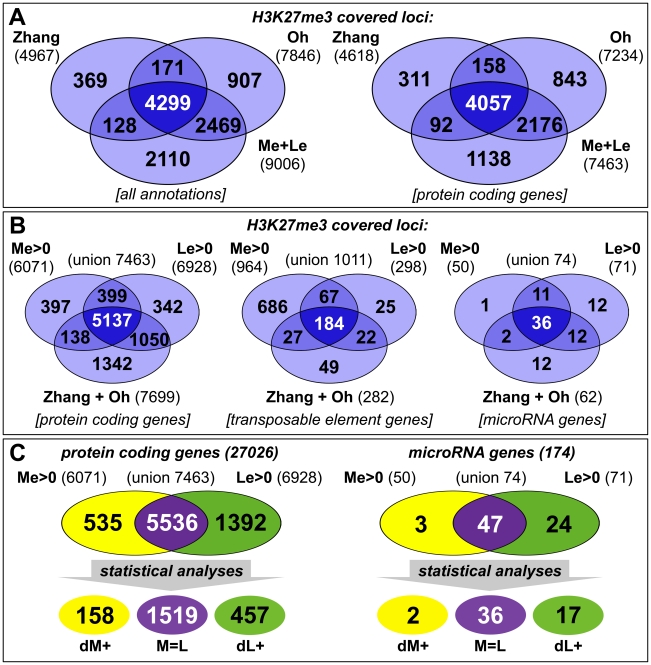
Tissue-specific ChIP-on-chip analysis reveals differentially methylated genes. (A, B) The venn diagrams represent a comparison of H3K27me3 covered loci taken from three independent genome wide analyses of *Arabidopsis* plants (Zhang *et al*. [5]; Oh *et al*. [20] and this work, meristem (Me) + leaves (Le)). (A) Comparison of all annotations (left) and of protein coding genes (right) in published data and union of Me+Le. (B) Comparison of protein coding (left), transposable element (middle) and microRNA genes (right) in union of published data and Me and Le sample. (C) Comparison of Me and Le targets, protein coding (left) and microRNA genes (right). Statistical analyses (see methods) were applied to uncover significantly differentially or equally methylated genes in meristem and leaves. Annotations are based on TAIR8 genome annotation.

It was previously suggested that H3K27me3 is required for stable gene repression throughout most of the plants lifecycle and is either reset to allow gene activation or acquired at later developmental stages to confer stable expression states during differentiation processes [5], [34]. In order to uncover dynamic regulation of H3K27me3 during development, undifferentiated meristematic (Me) and differentiated leaf (Le) tissues of *clavata3* (*clv3*) mutant plants were analysed. Meristematic tissue and stem cells can be easily isolated from *clv3* mutants by manual dissection as the mutant harbours larger vegetative shoot apical meristems and increased stem cell numbers [35] (Figure 1A). Importantly, leaf development is not affected by loss of *CLV3*. Both leaf and meristematic tissue samples were subjected to array based genome wide expression (Figure 1) and H3K27me3 (Figure 2) profiling. Therefore, this approach permits the identification of H3K27me3 targets that gain or lose H3K27me3 during differentiation of meristematic cells. In addition, the associated changes in gene expression of H3K27me3 targets can be revealed.

The expression analysis verified both sample identity and specific enrichment of each dissected tissue because typical transcripts characteristic for the meristematic domain or green tissue were detected as highly and differentially expressed (Figure 1C). Meristem and stem cell identity genes like *SHOOTMERISTEMLESS* (*STM*), *CLV3* and *WUSCHEL* (*WUS*) were highly expressed in the meristematic sample whereas photosynthesis-related genes encoding for subunits of RUBISCO (*RBCS-1B*) or light harvesting complexes (*LHCB1.4*) were exclusively detected in leaves.

We generated genome-wide H3K27me3 profiles for each tissue by hybridizing sheared genomic DNA that was immunoprecipitated with an antibody detecting H3K27me3 to high density tiling arrays, as was previously described [36], [37]. We identified a total of 9006 H3K27me3 target genes in the two tissues which include more than 7400 protein coding genes (27,6% of all annotated *Arabidopsis* protein coding genes) and interestingly also 74 miRNA genes (43% of all miRNA genes) (Figure 2). Importantly, more than 80% of protein coding H3K27me3 target genes identified in our study were also revealed in previous analyses of young seedlings [5], [20], demonstrating the validity of our approach. Both former studies were performed with 14 days old wildtype plants, whole seedlings or their aerial parts, whereas the tissue samples analyzed in our study were derived from 9 weeks old *clv3* mutant plants. The large H3K27me3 overlap in the different tissues therefore suggests extensive and stable gene silencing by Pc-G mediated H3K27me3 throughout vegetative plant development. In addition to a large number of common H3K27me3 targets, the analysis identified many, presumably tissue-specific protein coding target genes (1001 genes only detected in seedlings (by Oh et al. [20]); 1230 genes only identified in meristem or young leaf tissue in our study) (Figure 2A). Interestingly, although previous studies showed that transposable elements (TE) are largely devoid of H3K27me3 [5], [20], we detected a high number of TEs as meristem-specific H3K27me3 targets (Figure 2B). This suggests an unexpected role for Pc-G proteins in the regulation of TEs specifically in the stem cell harbouring meristem.

To study the role of H3K27me3 in controlling developmentally important genes we focused our analyses on protein coding and miRNA genes and compared the methylation profiles of the individual tissues (Figure 2B, 2C). This uncovered large differences between both samples as nearly 2000 protein coding and 27 miRNA genes were exclusively methylated in one sample (Me or Le) revealing these as tissue-specific H3K27me3 targets (details for all annotations are shown in Table S1). Interestingly, in contrast to the largely meristem-specific H3K27me3 targeting of TEs, a larger set of protein coding and miRNA H3K27me3 targets was identified in the leaf compared to the meristematic sample (Figure 2B). Thus, heterochromatic and euchromatic loci may be differentially targeted by Pc-G proteins in undifferentiated and differentiated tissues.

For further analyses on the comparison of H3K27me3 presence and gene expression and on gene families we isolated genes harbouring defined methylation levels and performed conservative statistical analyses to group these into sets of equally (M = L) and differentially methylated genes [meristem- (dM+) and leaf- (dL+) specific] (see methods). These analyses uncovered 1519 protein coding genes showing equal H3K27me3 levels in both samples, 158 genes specifically methylated in the meristem and 457 genes methylated in leaves (Figure 2C).

Collectively, our analyses uncover dynamic regulation of a large number of H3K27me3 target genes during differentiation which involves acquisition and removal of H3K27me3 at protein coding, miRNA and TE genes.

### Confirmation of differential methylation in wildtype meristems and leaves

**Figure 3:**
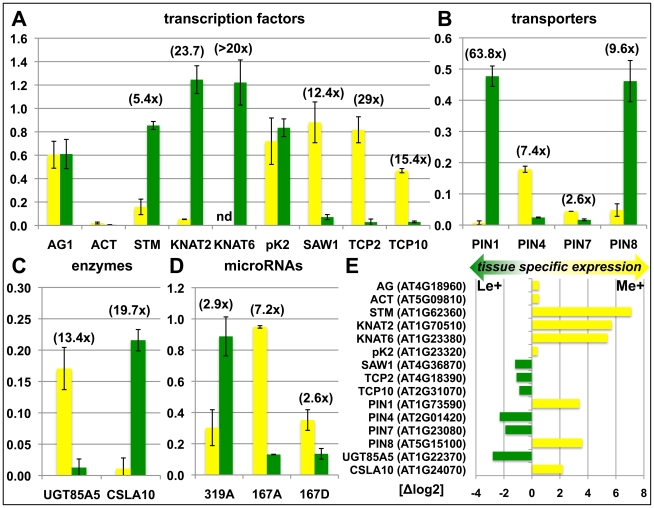
Confirmation of differentially methylated genes in wildtype plants. Differential H3K27me3 in meristem and leaves of wildtype was confirmed by independent H3K27me3-ChIP analyses for selected transcription factors (A), transporters (B), enzymes (C) and miRNA genes (D). Green: relative enrichment of H3K27me3 in leaves; yellow: relative enrichment of H3K27me3 in meristem. Enrichment factors between different tissues are indicated in brackets. 2 months old, short-day grown wild type plants were manually dissected. Enrichment of specific loci is normalized to the H3K27me3 target *FUSCA3* harbouring equal methylation levels in both tissues. The results are shown as mean values of two independent precipitations and qPCR analyses. Methylation of *KNAT6* was not detectable (nd) in the meristem sample. (E) Differential expression of genes analyzed in (A) – (D). Data was retrieved from whole transcriptome analyses of manually dissected *clv3* mutant meristems and leaves.

We sought to confirm the genome-wide, tissue-specific H3K27me3 patterns by independent ChIP experiments on meristems and leaves of *clv3* mutants (Figure S1) and wildtype (Figure 3). The transcription factors *KNOTTED-LIKE FROM ARABIDOPSIS THALIANA2* (*KNAT2*) and *KNAT6* are specifically expressed in the meristem ([38], (Figure 3)) and were identified as leaf specifically methylated (dL+) (Figure S1A representatively shows the *KNAT6* locus). H3K27me3 ChIP-qPCR analysis of independently dissected *clv3* tissues confirmed low meristematic and high leaf H3K27me3 levels for *KNAT2* and *KNAT6* and revealed an antagonistic pattern of the active mark H3K4me3 (Figure S1B, S1C). Thus, H3K4me3 and H3K27me3 distribution are consistent with meristem specific expression of both *KNAT* genes (Figure 3E). To further prove the significance of differential H3K27me3 identified by ChIP-chip on *clv3* mutants (Figure 2) we performed ChIP-qPCR analyses of 2 months old dissected wildtype plants, grown under short day conditions. Consistent with the data on *clv3*, *KNAT2* and *KNAT6* showed leaf specific H3K27me3 (Figure 3). In addition, equal methylation for the non-expressed genes *AGAMOUS* and *FUSCA3* and differential methylation of several transcription factor genes, *PIN-FORMED* (*PIN*) auxin efflux carriers and microRNA genes were confirmed (Figure 3). For all genes tested by ChIP, moderate to strong differential expression was detected which was highest in the tissues lacking H3K27me3 (Figure 3E). Thus, these analyses strongly suggest that the *clv3* ChIP-chip results are transferable to wild type plants.

### Presence of H3K27me3 is correlated with gene repression in a tissue-specific manner

**Figure 4:**
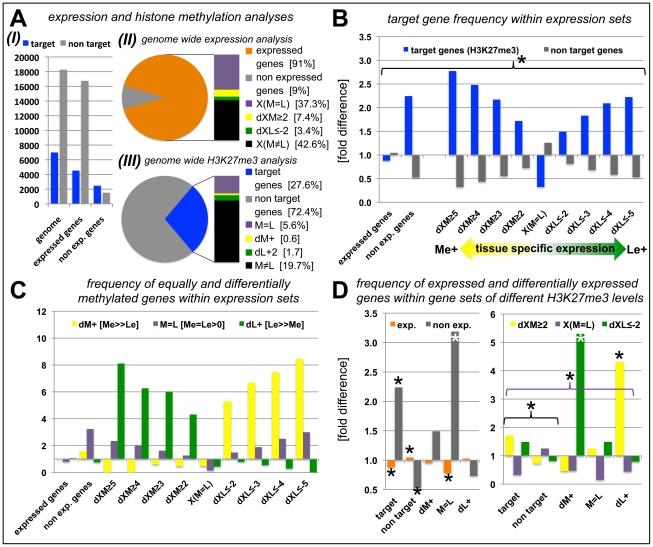
Anti-correlation of gene expression and repressive H3K27 tri-methylation. (A) Genome-wide relation of gene expression and H3K27me3 targeting (I). (II) Representation of percentage of genes for defined expression states. (III) Representation of percentage of genes for defined H3K27me3 levels. For expressed genes and H3K27me3 targets, number of genes detected in leaves or meristems of *clv3* mutants was merged and compared to genes present on Agilent array (for II) or all protein coding genes (for III). (B) Relative proportion of H3K27me3 target genes within gene sets of defined expression levels, y-axis indicates fold enrichment compared to subset frequency in the genome, i.e. target genes or non target genes. (C) Relative proportion of equally and differentially methylated H3K27me3 target genes within gene sets of defined expression levels, y-axis indicates fold enrichment compared to specific subset frequency in the genome. (D) Relative proportion of differentially or equally expressed genes within gene sets of differentially, equally or non-methylated genes, y-axis indicates fold enrichment compared to specific subset frequency in the genome. Significant differences (Students tTest; *p*≤0.05) are marked with an asterisk (*). Coloured brackets indicate significant differences for similarly coloured bars within the bracket. Statistical analyses and gene lists are shown in Table S2. Abbreviations: exp. (expressed); dXM (preferential expression in meristem as Δlog2(Me/Le)); dXL (preferential expression in leaf as Δlog2(Me/Le)); X(M = L) (equal expression in meristem and leaf); dM+ (H3K27me3 meristem specific); dL+ (H3K27me3 leaf specific); M = L (equal level of H3K27me3 in leaf and meristem); see methods for details on subsets of differentially expressed and methylated genes.

Since we had identified tissue-specific H3K27me3 target genes we asked whether differential H3K27me3 is correlated with differential gene expression. In total, more than 22000 protein coding genes (85% of the genome) were expressed in at least one of the tissues. A large fraction (nearly 9500 genes) showed equal expression in both samples [X(M = L)], whereas 2.833 were at least four times higher expressed in one sample compared to the other [1.865 higher in the meristem (dXM≥2, Δlog2) and 968 higher in leaves (dXL≤−2, Δlog2)] (Figure 4A, Figure S2). We next binned genes with similar expression differences in the two tissues (Figure 4B, Table S2). These gene sets were analyzed for enrichment of H3K27me3 targets (Figure 4B) and of genes harbouring equal or differential H3K27me3 levels (M = L, dM+, dL+) (Figure 4C). In addition, we performed the reciprocal analyses (Figure 4D). Genes that were equally expressed both in the meristem and the leaves (X(M = L)) showed a significant underrepresentation of H3K27me3 targets (Figure 4B). In contrast, methylated genes were more frequent in the groups of non expressed and differentially expressed genes (Figure 4B). Interestingly, the relative abundance of target genes increased with increasing tissue specific expression in the meristem (Me+) or leaves (Le+) (Figure 4B). We next asked how differential expression relates to differential or equal H3K27me3 (Figure 4C). This detailed tissue specific analysis clearly revealed that the highly specific expression in one tissue (dXM≥2 or dXL≤−2) was correlated with a depletion of tissue specific methylation (dM+ or dL+) in the same sample and a strong enrichment of repressive histone methylation in the other – non expressing – sample. Importantly, the stronger the differences in gene expression levels between the two tissues, the more genes were identified which showed antagonistic, differential H3K27me3 (Figure 4C). Genes showing equal H3K27me3 levels in both tissues were enriched for non-expressed genes which was not the case for differentially methylated genes, revealing that most genes carrying differential methylation are expressed in at least one of the tissues (Figure 4C, 4D). We found the same correlations when the analyses were performed with the different subsets of H3K27me3 covered genes (Figure 4D). Leaf-specifically expressed genes (dXL≤−2) were more frequent in the set of meristem specifically methylated genes (dM+) and *vice versa* whereas genes showing equal, but detectable methylation (M = L) were depleted of expressed genes.

In whole genome analyses of H3K27me3 in seedlings, Oh et al. [20] identified 1001 H3K27me3 targets which were not revealed in our analyses (Figure 2A). 824 of these are expressed in at least one of the tissues we analyzed (data not shown), suggesting that these targets are seedling-specifically methylated and are subjected to dynamic changes of H3K27me3 in development.

### Functional characterization of H3K27me3 target genes by Gene Ontology and gene family analyses

Previous analyses of H3K27me3 target genes in seedlings identified transcription factors as a major class of H3K27me3 target genes [5]. To reveal whether all transcription factor families and other gene families are preferentially targeted by H3K27me3 we analyzed our datasets generated from leaves and meristems.

We first exploited the resources of the Gene Ontology (GO) annotations from TAIR (http://www.arabidopsis.org/) providing functional categorizations of *Arabidopsis* protein coding genes (Figure S3). As expected the molecular function “transcription factor activity” was enriched in H3K27me3 target genes, whereas general cellular housekeeping functions like “DNA or RNA metabolism” and “electron transport or energy pathways” were depleted. Importantly, also tissue-specific differences were uncovered: a significant depletion of leaf-specific H3K27me3 target genes was observed in the cellular component “plastid”, consistent with the restriction of photosynthesis to leaves. In addition, genes associated with “kinase activity” and “transporter activity” were revealed as preferential H3K27me3 targets in the meristematic sample, suggesting specific repression of these sets of genes in undifferentiated cells.

**Figure 5:**
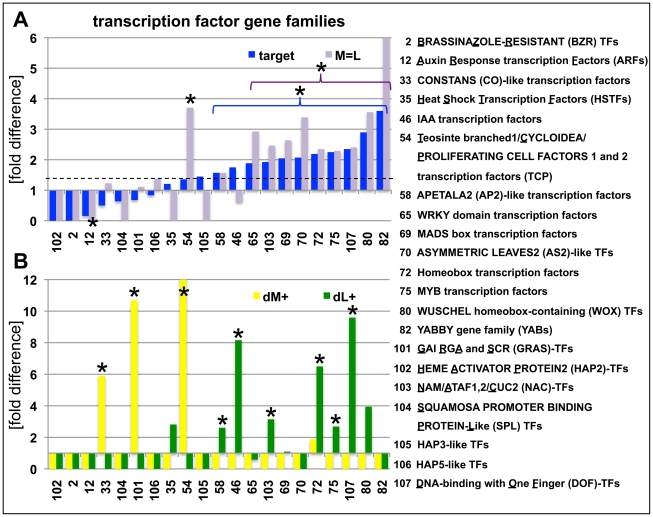
Abundance of H3K27me3 target genes in transcription factor gene families. (A) Relative enrichment or depletion of H3K27me3 target genes (blue) or equally methylated genes (M = L, purple) in specific transcription factor families, y-axis indicates fold difference relative to the observed frequencies of specific subsets within the genome. The dashed line indicates relative enrichment of H3K27me3 targets in all transcription factor genes (1907 in total, taken from (http://plntfdb.bio.uni-potsdam.de/v3.0/)) compared to genome average. (B) Relative enrichment or depletion of meristem-specific (dM+, yellow) or leaf-specific (dM+, green) H3K27me3 target genes in specific transcription factor families, y-axis indicates fold difference relative to the observed frequencies of specific subsets within the genome. Asterisks indicate significant differences (*p*≤0.05, χ^2^-test) compared to total number of genes for a specific subset. Coloured brackets indicate significant differences for similarly coloured bars within the bracket. Details of statistical analyses can be found in Table S3. Analyses of additional gene families are shown in Figure S4.

We further dissected the H3K27me3 target genes by detailed analysis of gene families with a focus on transcription factor, enzyme and transporter gene families as these were enriched in the broad GO term analyses of H3K27me3 targets (Figure 5; Figure S4). Gene families involved in fundamental cellular processes like DNA replication, cell cycle regulators and protein phosphorylation/dephosphorylation were significantly depleted in H3K27me3 target genes (Figure S4; Table S3). Surprisingly, several transcription factor families were largely not regulated by H3K27me3 including the *AUXIN RESPONSE FACTOR* (*ARF*), *CCAAT-HAP2* (*HAP2*) and *SQUAMOSA-PROMOTER BINDING LIKE* (*SPL*) gene families (Figure 5). Other transcription factor gene families showed a strong bias towards tissue-specific targeting by H3K27me3 (dM+; dL+) suggesting a specific regulation in the meristem or leaves. Leaf-specific H3K27me3 target genes comprise known regulators of meristem function like *HOMEOBOX* transcription factors and several other gene families (e.g. *IAA* and *DOF* transcription factors), while *TCP*, *CONSTANS*-like and *GRAS*-transcription factors were targeted by H3K27me3 specifically in the meristem indicating a role for theses gene families in differentiation processes or leaf development. A surprisingly large set of enzyme and transporter gene families were uncovered as H3K27me3 targets which are involved in differentiation or developmental processes (e.g. cell wall biosynthesis), hormone biosynthesis (YUCCA flavin monooxygenases, CYTOCHROME P450s) or transport of photosynthetic products (sucrose transporters) and hormones (PINFORMED (PIN)-like auxin efflux carriers) (Figure S4, Table S3). Previous analysis had identified the actin regulator formin ARABIDOPSIS FORMIN HOMOLOGUE 5 (AtFH5) as Pc-G target gene whose mis-expression is partially responsible for Pc-G mutant seed phenotypes, revealing that regulation of enzymatic genes is an important function of Pc-G proteins [39].

Lastly, we studied whether specific transposable element gene families were overrepresented in the different tissues. We generally observed a significantly larger amount of retrotransposons compared to DNA transposons as H3K27me3 target genes (Figure S4H, Table S3). Also tissue-specific differences were identified among retrotransposon gene families. Whereas gypsy-like retrotransposons were significantly enriched in the meristematic fraction, copia-like retrotransposons were overrepresented in leaves and athila-like retrotransposons almost completely non-targeted by H3K27me3 in leaves (Figure S4H).

Collectively, these analyses uncovered H3K27me3 targeting of a diverse set of gene families which are involved in biosynthesis and transport of secondary metabolites and transcriptional regulation and ultimately in cellular differentiation. These sets of genes include many known regulators of meristem or leaf development and provide an important resource for the identification of additional, novel factors involved in differentiation or stem cell regulation. In addition, also specific transposable element gene families are tissue-specifically targeted by H3K27me3 suggesting differential regulation of TE genes in the different tissues.

### H3K27me3 targets specific miRNA gene families and is reduced at miRNA target genes

During the analyses of H3K27me3 targets we found that a large number of miRNA and several trans-acting siRNA (tasiRNA) genes carried H3K27me3 which was, to our knowledge, not previously revealed (Figure 2). These regulatory RNAs display differential methylation in the same way as protein coding genes, thus many show leaf-specific H3K27me3, whereas only few carry H3K27me3 exclusively in the meristem (Figure 2C). Seventy four of the annotated 174 miRNA loci were identified in our analysis and we revealed additional 12 that were detected, but not described by previous studies [5], [20]. Thus, nearly 50% of all microRNA genes are covered by H3K27me3 (Figure 2, Table 1). A high number of miRNAs (25 of 103 unique miRNAs) are encoded in multiple loci which constitute 96 of all 174 miRNA loci (*Arabidopsis* Small RNA Project (http://asrp.cgrb.oregonstate.edu/) [40]). Multiple and single loci miRNAs are regulated by H3K27me3 but miRNAs encoded in multiple loci are heavily targeted (72%) whereas only 22% of miRNAs present at a single locus are H3K27me3 targets.

**Table 1:** Regulation of miRNA and tasiRNA genes and their targets by H3K27me3.

type of small RNA	no. of genes		
	total	H3K27me3 targeted	
miRNAs, single locus [78]	78	17	21.8%
miRNAs, multiple loci [25]	96	69	71.9%
***total***	***174***	***86***	***49.4%***
tasiRNAs [4]	8	2	25.0%

protein coding genes regulated by	no. of genes		
(type of small RNA)	total	H3K27me3 targeted	
miRNAs (H3K27me3 targeted)	90	31	34.4%
miRNAs (non targeted)	119	49	41.2%
***total***	***206***	***78***	***37.9%***
tasiRNA	43	11	25.6%

H3K27me3 targeted small regulatory RNA genes encoded at single or multiple sites in the genome. H3K27me3 targets among miRNA or tasiRNA regulated protein coding genes.

Currently 206 genes including many developmentally important transcription factors are known which are negatively regulated by miRNAs at a post-transcriptional level. Interestingly, genes that are regulated by a H3K27me3 covered miRNA genes less frequently carry H3K27me3 compared to genes whose expression is controlled by a miRNA gene devoid of H3K27me3 (Table 1). This bias is even more apparent when specific miRNA and tasiRNA gene families and their targets are analyzed (Table 2): whereas most *miRNA169*, *miRNA156/157*, *miRNA167* and *tasiRNA3* loci carry H3K27me3, their target genes (*HAP2*, *SPL* or *ARF* genes) are largely devoid of H3K27me3. The lack of H3K27me3 targeting of these transcription factor genes is in contrast to the generally high enrichment of transcription factors as H3K27me3 targets (Figure 5; compare *HAP2*, *SPL* and *ARF* families to average enrichment in H3K27me3 targets of all transcription factor genes, p<0.05 (χ^2^-test) for *HAP2* and *ARF* genes (Table S3)). However, H3K27me3-mediated repression of the miRNA genes likely permits an indirect positive regulation of these specific transcription factor families by Pc-G proteins. Interestingly, for other gene families (e.g. *TCP* and *HD-ZIP* transcription factors) both miRNA genes and their targets carry H3K27me3, suggesting that expression of these transcription factors is regulated transcriptionally by Pc-G proteins and post-transcriptionally by miRNAs.

**Table 2:** H3K27me3 targeted miRNAs or tasiRNAs encoded by multiple loci and H3K27me3 regulation of their target genes.

small RNA	no. of genes			target gene family	no. of genes		
(name)	total	H3K27me3		(name)	miRNA	H3K27me3	
		targeted			targeted		
miR394	2	2	100%	F-Box	1	0	0%
miR845	2	2	100%	-	-	-	-
miR164	3	3	100%	NAC	7	2	29%
miR158	2	2	100%	PPR	22	3	14%
miR166/165	9	8	89%	HDZIPIII	5	2	40%
miR169	14	12	86%	HAP2	7	0	0%
miR395	6	5	83%	APS,AST	4	1	25%
miR399	6	5	83%	E2-UBC	1	0	0%
miR156/157	12	10	83%	SPL	11	1	9%
miR172	5	4	80%	AP2	6	2	33%
miR160/167	7	5	71%	ARF	5	0	0%
miR159/319	6	4	67%	MYB/TCP	12	5	42%
miR171	3	2	67%	SCL	3	1	33%
miR393	2	1	50%	TIR1 (F-box)	5	0	0%
miR168	2	1	50%	AGO	1	0	0%
miR397	2	1	50%	LAC	3	2	67%
miR390	2	1	50%	TAS3	3	2	67%
miR447	3	1	33%	2-PGK	2	1	50%
***total***	***88***	***69***	***78%***	***total***	***98***	***22***	***22%***
tasiRNA3	3	2	67%	ARF	3	0	0%

All 174 annotated miRNA loci (based on TAIR8 annotation) were analyzed, 74 of them are H3K27me3 targeted in our analyses and 12 additional loci were identified as targets by Oh *et al*. [20]. miRNA regulated genes were extracted from the *Arabidopsis* Small RNA Project (http://asrp.cgrb.oregonstate.edu/) and TAIR (www.arabidopsis.org).

### Biosynthesis, transport, perception, and signaling genes of the phytohormone auxin are regulated by Pc-G/H3K27me3

Our in-depth gene family analyses revealed enrichment of specific biosynthetic enzymes, transporters and transcription factors (Figure 5). Therefore, we inquired whether H3K27me3 targets entire developmental pathways which involve local biosynthesis of a signaling molecule, its transport, perception and signal transduction. We focused on the phytohormone auxin as we initially realized that the PIN auxin efflux carriers are largely H3K27me3 targets (Figure S4). Several pathways and gene families (*YUCCA* monooxygenases, *CYTOCHROME P450s* and *TRYPTOPHANE AMINO TRANSFERASES*) participate in the biosynthesis of the phytohormone auxin from tryptophan (reviewed in [41], [42]). Auxin is transported by several different carrier proteins in the plant. The *AUXIN RESISTANT1* (*AUX1*)/*LIKE AUX1* (*LAX*) gene family enables auxin influx and *PIN*- and several *ATP*-*BINDING CASSETTE* (*ABC*) transporters mediate auxin efflux (reviewed in [43]). The analysis of gene families uncovered that most of these gene families are enriched for H3K27me3 targets (Figure 5, Figure S4), including the genes for which a role in auxin regulation was shown (Table 3, Table 4). Interestingly, although the auxin receptor gene *TRANSPORT INHIBITOR RESPONSE 1* (*TIR1*) and its homologues are largely devoid of H3K27me3, *miRNA393a* which negatively regulates these is targeted by H3K27me3 in leaves (Table 4). Similarly, the *ARF* transcription factor genes which are involved in repressing auxin inducible genes (reviewed in [44]) are depleted in H3K27me3, however, miRNAs and tasiRNAs controlling *ARF* expression levels are largely targeted by H3K27me3 (Figure 5, Table 2, Table 4). On the other hand, *IAA* class of transcription factors which positively regulate auxin responsive genes are highly enriched for H3K27me3 targets, similar to other transcription factor families. Thus, auxin biosynthesis, transport, perception and transcriptional responses are controlled by H3K27me3, both by direct targeting of the regulatory genes or by indirect regulation through miRNAs. The analyses of the auxin pathway therefore reveal that not only specific gene families but also entire pathways involving diverse gene families can be controlled by H3K27me3.

**Table 3:** Genes involved in auxin biosynthesis and transport are regulated by PcG/H3K27me3.

*source (biosynthesis and transport)*			
gene family/gene (name)	no. of loci (accession)	H3K27me3 targeted loci	
* biosynthetic enzymes:*			
**YUCCAs**	**34**	**23**	**67.6%**
YUC1	AT4G32540	+	
YUC2	AT4G13260	M = L	
YUC4	AT5G11320	+	
YUC6	AT5G25620	+	
YUC11	AT1G21430	−	
**Cytochrome P450s**	**227**	**147**	**64.8%**
CYP79b2	AT4G3995	+	
CYP79b3	AT2G22330	+	
CYP83b1	AT4G31500	−	
CYP71A13	AT2G30770	M = L	
**TRP-a-transferases**	**3**	**2**	**66.7%**
TAA1	AT1G70560	+	
TAR1	AT1G23320	M = L	
TAR2	AT4G24670	−	
**other enzymes**			
SUR1 (C-S LYASE)	AT2G20610	−	
NIT1 (Nitrilase)	AT3G44310	−	
NIT2 (Nitrilase)	AT3G44300	+	
NIT3 (Nitrilase)	AT3G44320	−	
* transporters:*			
**PINs**	**8**	**5**	**62.5%**
PIN1	AT1G73590	dL+	
PIN2	AT5G57090	M = L	
PIN3	AT1G70940	−	
PIN4	AT2G01420	(+*)	
PIN5	AT5G16530	M = L	
PIN6	AT1G77110	dL+	
PIN7	AT1G23080	−	
PIN8	AT5G15100	+	
**AUX/LAXs**	**4**	**3**	**75.0%**
AUX1	AT2G38120	dL+	
LAX1	AT5G01240	−	
LAX2	AT2G21050	+	
LAX3	AT1G77690	dL+	
**ABCs**	**128**	**46**	**35.9%**
* (ABCb1; ABCb4 and ABCb19 are no targets)*			

The table shows the fraction of H3K27me3 targeted genes in gene families involved in auxin biosynthesis and transport. Genes described to be involved in auxin biosynthesis [41], [42] or transport [43], [64] are listed below the corresponding gene family. (+*) *PIN4* was not detected as H3K27me3 target by ChIP-chip but by ChIP-qPCR in wildtype meristems (see Figure 3).

**Table 4:** Genes involved in auxin perception and signaling are regulated by PcG/H3K27me3.

*sink (perception and signaling)*			
gene family/gene (name)	no. of loci (accession)	H3K27me3 targeted loci	
* receptors:*			
**TIR1/Fboxes**	**6**	**1**	**16.7%**
TIR1	AT3G62980	−	(miR393)
AFB1	AT4G03190	−	(miR393)
AFB2	AT3G26810	+	(miR393)
AFB3	AT1G12820	−	(miR393)
AT4G24390	AT4G24390	−	(−)
AT5G49980	AT5G49980	−	(−)
* transcription factors:*			
**IAAs**	**29**	**14**	**48.3%**
IAA1	AT4G14560	dL+	
IAA2	AT3G23030	dL+	
IAA5	AT1G15580	+	
IAA6	AT1G52830	M = L	
IAA7	AT3G23050	+	
IAA14	AT4G14550	+	
IAA15	AT1G80390	+	
IAA17	AT1G04250	+	
IAA19	AT3G15540	+	
IAA20	AT2G46990	dL+	
IAA28	AT5G25890	+	
IAA29	AT4G32280	+	
IAA30	AT3G62100	dL+	
IAA33	AT5G57420	+	
**ARFs**	**23**	**1**	**4.3%**
ARF2	AT5G62000	−	(tasiR3)
ARF3	AT2G33860	−	(tasiR3)
ARF4	AT5G60450	−	(tasiR3)
ARF6	AT1G30330	−	(miR167)
ARF7	AT5G20730	+	(−)
ARF8	AT5G37020	−	(miR167)
ARF10	AT2G28350	−	(miR160)
ARF16	AT4G30080	−	(miR160)
ARF17	AT1G77850	−	(miR160)

The table shows the fraction of H3K27me3 targeted genes in gene families involved in auxin perception or signaling. Genes described to be involved in auxin perception [65] and the regulation of auxin dependent transcription [44] are listed below the corresponding gene family. Several members of *TIR1* and *ARF* families are regulated by small RNAs, therefore also H3K27me3 targeted miRNA or tasiRNA genes are depicted.

### H3K27me3 is entirely dependent on Pc-G proteins

**Figure 6:**
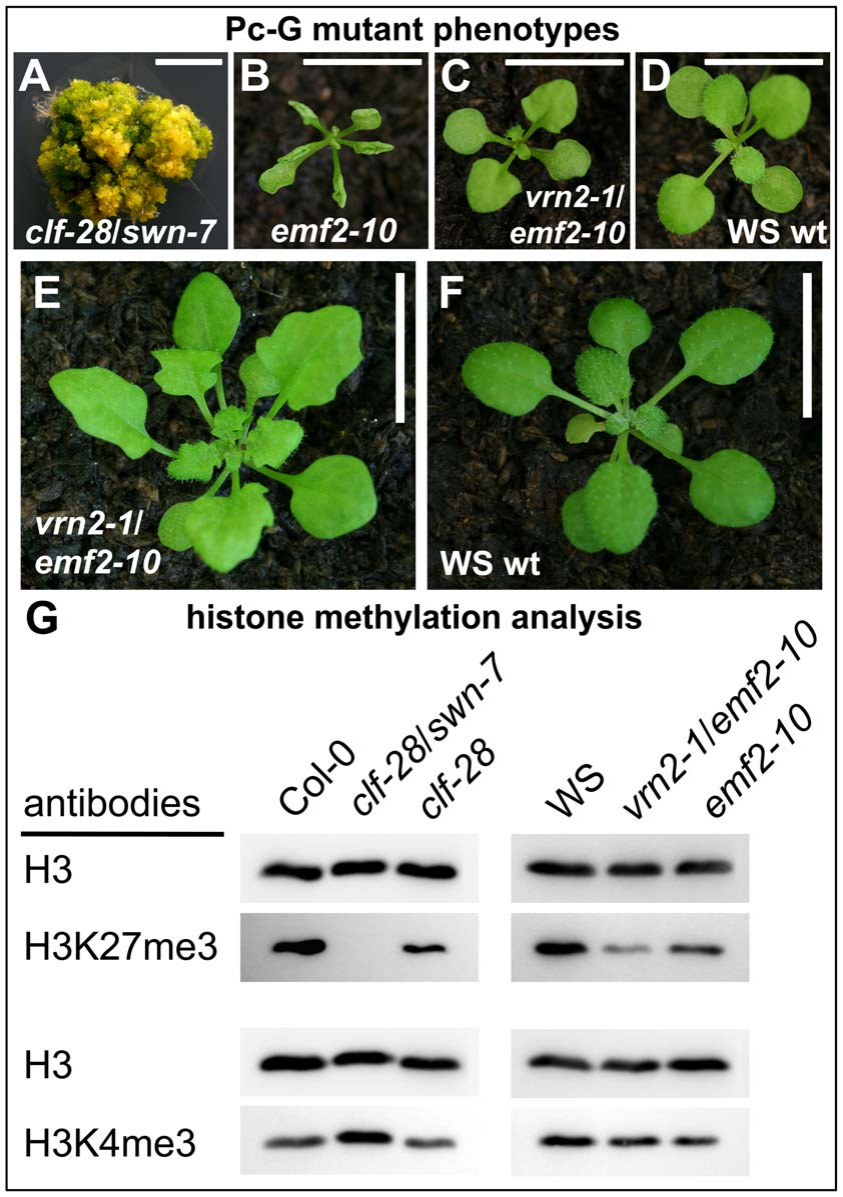
Immunoblot analyses of histone modifications in Pc-G mutants. (A) *clf-28*/*swn-7* double mutants, 60 d old and grown in axenic conditions in long days, (B) *emf2*–*10,* (C) *vrn2*–*1/emf2*–*10* double mutants, (D) Wassilewskija (WS) wildtype. Plants in (B)–(D) are 17 old and grown in short day conditions. (E), (F) 45 d old plants grown under short day conditions, (E) *vrn2-1*/*emf2-10* double mutant, (F) wildtype. Scale bars in pictures (A) to (D) indicate 0.5 cm and in (E) and (F) 1 cm. (G) Immunoblot analyses of H3K4me3 and H3K27me3 levels in Pc-G mutants. Similar amounts of histone H3 were loaded as confirmed with a H3 specific antibody detecting the unmodified C-terminal part of H3. Immunoblot analyses of additional histone modifications are shown in Figure S5.

Only selected plant genes expressed in vegetative tissues were previously analyzed for an overlap of H3K27me3 and Pc-G protein binding and dependence of H3K27me3 on Pc-G proteins [8], [14], [45], [46]. Although immunofluorescence analyses on the severe *clf/swn* mutants revealed a reduction in euchromatic H3K27me3 [15], it is currently unclear whether the H3K27me3 is completely dependent on Pc-G proteins. To reveal whether presence of H3K27me3 is likely corresponding to Pc-G binding and regulation we used immunoblot analyses to study histone methylation levels in various PRC2 mutants. We used mutants that are partially or completely deficient for *Arabidopsis* homologs of *Drosophila* E(z) [*clf-28* (null allele), *swn-7* (null allele)] or Suppressor of zeste 12 [*vernalization2-1 (vrn2-1)* (null allele), *embryonic flower2*–*10* (*emf2*–*10)* (weak allele of *emf2*)] [18], [47]. We studied single and double mutants of these as they show different degrees of reduction in somatic Pc-G activity: moderate reduction in *clf-28 and emf2*–*10* more severe reduction in *vrn2/emf2*–*10* double mutants and complete loss of somatic Pc-G activity in the *clf/swn* double mutant (Figure 6) [18]. H3K27me3 was completely lost in the *clf*/*swn* mutants, strongly reduced in *vrn2/emf2-10* mutants and only mildly affected in *clf* or *emf2*–*10* mutants (Figure 6). Loss of H3K27me3 in *clf*/*swn* was correlated with a strong increase in H3K4me3. H3K27me1, a modification preferentially present in heterochromatin, was not affected in any of the mutants whereas H3K27me2 showed a clear reduction in *vrn2/emf2*–*10,* but increase in *clf*/*swn* mutants (Figure S5). Thus, our study of histone methylation in Pc-G mutants strongly suggests that all H3K27me3 is catalyzed by PRC2 proteins and that a possible re-distribution to heterochromatic regions [15] involves only marginal amounts of H3K27me3.

Since the global analysis of H3K27me3 abundance revealed a strong reduction even in the *vrn2*/*emf2*–*10* double mutant showing relatively mild morphological alterations (Figure 6A) we were interested if a H3K27me3 reduction occurs at most genes or gene-specifically. We therefore studied H3K27me3 of selected genes in leaves from wildtype and *vrn2*/*emf2*–*10* plants by ChIP-qPCR (Figure S6). This analysis identified three different classes: *STM* and *FUS3* displayed no difference in H3K27me3 in the mutants, several genes including *KNAT2* or *PIN8* had intermediate H3K27me3 levels and most analyzed genes (e.g. *PIN1* and *PIN6*) showed a strong reduction or a complete loss in the mutants. Thus, the observed global reduction in H3K27me3 is rather caused by the loss of H3K27me3 at some loci than by an equal reduction of H3K27me3 at all target loci (Figure S6).

### Pc-G proteins control tissue-specific expression of their target genes

**Figure 7:**
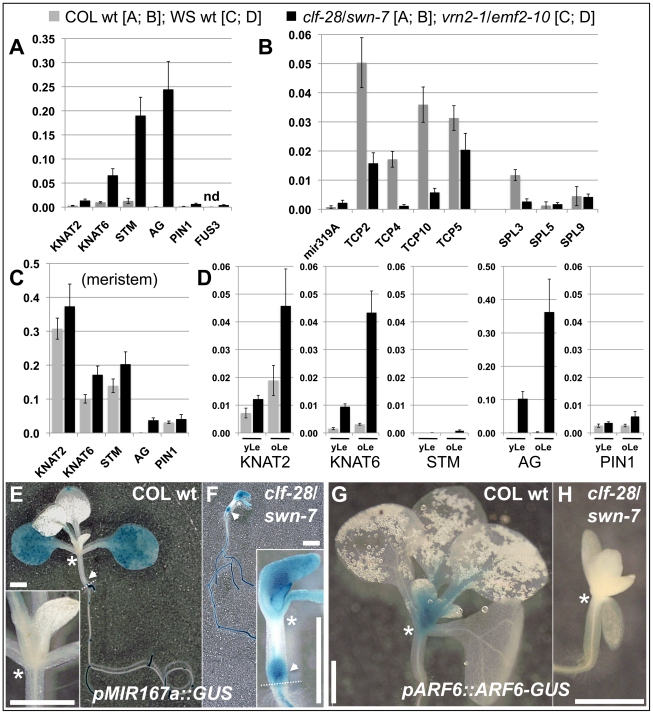
Pc-G proteins control tissue-specific expression of H3K27me3 target genes. (A) – (C) qRT-PCR based expression analyses of (A, B) 9 d old wildtype and *clf-28*/*swn-7* plants or (C, D) 45 days old, dissected wildtype and *vrn2-1*/*emf2*–*10* plants. Samples were enriched for meristem (Me) (C), young leaves (yLe, up to 3 mm blade length) or older leaves (oLe, 3 – 10 mm) (D). Expression values were normalized to At5g60390 (EF-1α). (E, F) Colorimetric analyses of *pMir167a::GUS* expression in 9 d old wildtype (E) and *clf-28/swn-7* double mutants (F). Insets are close-ups. (G, H) Expression of *pARF6::ARF6-GUS* 9 d old wildtype (G) and *clf-28/swn-7* double mutants (H). *pARF6::ARF6-GUS* carries the *mir167* binding site. Asterisks mark meristematic region, white arrow base of hypocotyl. The border between the hypocotyl and the root is indicated with a dashed white line. Scale bars indicate 1 cm. All plants were grown in short day conditions.

**Figure 8:**
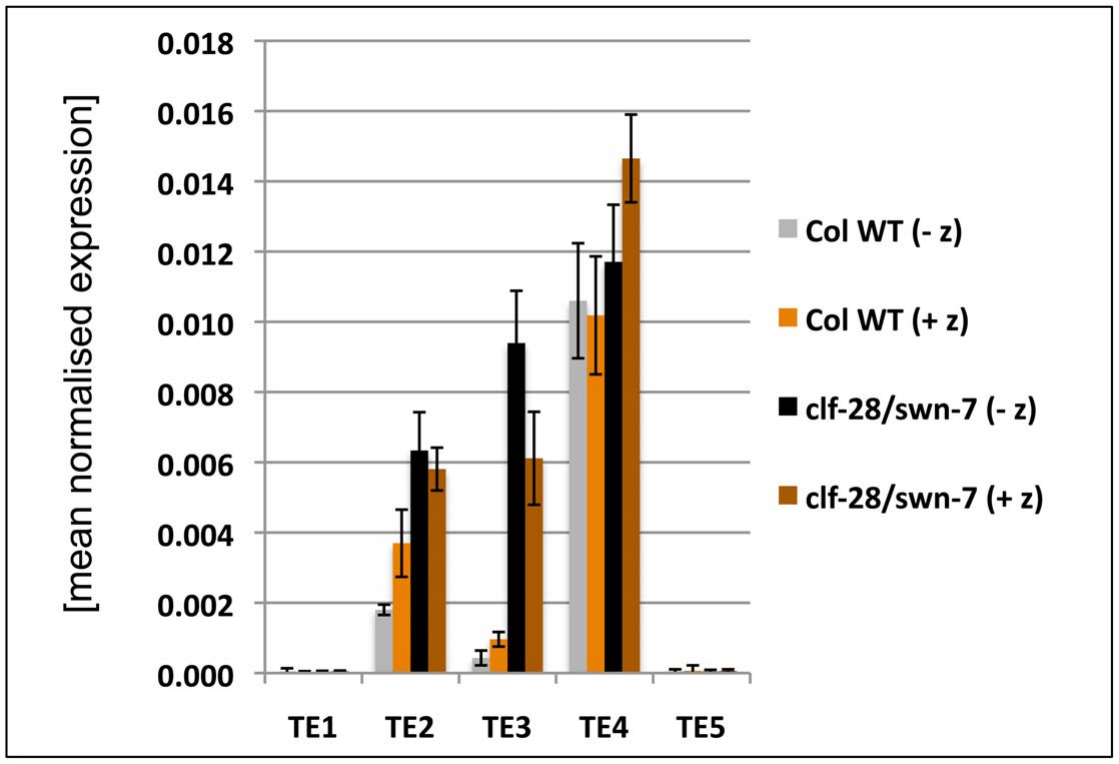
Expression analysis of transposable element genes. The expression of several transposable element genes was analysed in 9 d old wildtype and *clf-28/swn-7* seedlings by qRT-PCR. Plants were grown on media with (+ z) and without the DNA methyltransferase inhibitor zebularine (− z). Expression values were normalized to At5g60390 (EF-1α). [TE1 (AT3G28400; hAT transposase), TE2 (AT3G21020; copia element), TE3 (AT3G21040; copia element), TE4 (AT3G43690; copia element), TE5 AT4G02960; copia element)].

To study possible changes in gene expression resulting from a reduction in H3K27me3, we performed qRT-PCR based expression analyses of *clf*/*swn* and *vrn2/emf2*–*10* double mutants (Figure 7, Figure 8). *Clf/swn* mutants show complete loss of H3K27me3, do not maintain organ identity post-embryonically and are therefore only comparable to wildtype shortly after germination. However, *vrn2/emf2*–*10* mutants show strong leaf serration especially in older leaves, but produce leaves and flowers (Figure 6), thus tissue-specific changes in gene expression can also be analyzed at later developmental stages. In 9 d old *clf/swn* seedlings, we observed mis-expression of several previously analysed H3K27me3 targets [8], [48], [49], ranging from mild (*KNAT2*) to strong (*KNAT6*, *STM* and *AG*) mis-expression (Figure 7A). Surprisingly, for *TCP2, TCP4*, *TCP10* and *SPL3* we observed a strong down-regulation in *clf/swn* mutants although they likely lost H3K27me3 (Figure 7B). However, *TCP2, TCP4 TCP10* are negatively regulated by *miRNA319* [50] which is targeted by H3K27me3 (Figure 3). Consistently, we observed mis-expression of the *miRNA319a* precursor in *clf/swn* mutants which is likely responsible for post-transcriptional down-regulation of *TCP2*, *4* and *10* in the Pc-G mutant. Expression of *TCP5* which is not targeted by *miRNA319* is not affected in the mutant. Similarly, *SPL3* whose abundance is regulated by the H3K27me3 targeted miRNA156 showed reduced expression in *clf/swn* mutants (Figure 7B).

H3K27me3 targets 6 of the 8 PIN gene members (Table 3), we therefore also analysed their expression in *clf/swn* and *vrn2/emf2*–*10* mutants. Except for *PIN1*, none of the PIN genes showed upregulation in the mutants (Figure 7A, Figure S6). *PIN3*, *PIN4* and *PIN7* which are all not targeted by H3K27me3 in leaves even showed a strong down-regulation of expression in *clf*/*swn* mutants.

As we also revealed H3K27me3 targeting of several TE genes, we studied expression of several H3K27me3 targeted TE genes. We observed upregulation of two TE genes in the *clf/swn* mutant (Figure 8), suggesting that Pc-G proteins and H3K27me3 can prevent expression of TE genes, which is consistent with a previous study revealing Pc-G mediated silencing of a TE gene in the endosperm [51]. To study whether interference with DNA methylation can further enhance activation of TE genes, we grew wildtype and *clf/swn* plants on media containing the DNA methyltransferase inhibitor zebularine [52] and analysed TE activation (Figure 8). The tested TE genes were neither activated by zebularine in wildtype nor further transcriptionally activated in *clf/swn* suggesting DNA methylation independent regulation of the TE genes which are activated in the Pc-G mutants.

Lastly, we investigated whether Pc-G mutants control tissue-specific expression of differentially methylated H3K27me3 targets. *Mir167a* and *167d* genes show higher levels of H3K27me3 in the meristem compared to leaves (Figure 3). Reporter transgenes in which *β-GLUCURONIDASE (GUS)* was fused to the promoters of *mir167a* and *mir167d* [53] revealed preferential expression of the miRNA genes in cotyledons, but exclusion from the meristem, consistent with the H3K27me3 patterns (Figure 7, Figure S8). In *clf/swn* mutants, the expression domains of *mir167a* and *mir167d* reporters were expanded to the shoot apical meristem, the petioles, the main root and the base of the hypocotyl (Figure 7F, Figure S8). The genes *ARF6* and *ARF8* genes are regulated by *mir167* which is in agreement with their mutually exclusive expression domains [53] (Figure 7E, 7G). Consistent with mis-expression of *mir167* in *clf/swn* mutants we observed loss of shoot meristematic expression of a *pARF6::ARF6-GUS* reporter gene construct that harbours the miRNA binding site (Figure 7H). This further demonstrates the expansion of the miRNA expression domain to the meristem.

To uncover tissue-specific mis-expression of differentially methylated H3K27me3 targets in the *vrn2/emf2*–*10* mutant we dissected plants and analyzed gene expression in meristematic tissue, young leaves (up to 3 mm blade length) and older leaves (3–10 mm). In wildtype, *KNAT2*, *KNAT6*, *STM* and *PIN1* are strongly expressed in the meristem and carry H3K27me3 preferentially in leaves (Figure 3, Figure 7). Expression of these genes was not altered in meristems of *vrn2*/*emf2*–*10* double mutants as expected (Figure 7C). In older leaves of the mutant, however, the *KNAT* genes were activated which correlates with a reduction in H3K27me3 (Figure 7D, Figure S6). *STM* displayed only moderate mis-expression in leaves, consistent with similar levels of H3K27me3 in wildtype and *vrn2*/*emf2*–*10* mutants. Although H3K27me3 of *PIN1* is depleted in *vrn2*/*emf2-10* mutant leaves the gene is only slightly activated in the mutant (Figure 7, Figure S6). For several H3K27me3 target genes exhibiting differential methylation we revealed tissue-specific mis-expression in Pc-G mutants indicating that Pc-G proteins restrict expression of their targets by depositing tissue-specific H3K27me3. We also showed that Pc-G proteins transcriptionally regulate miRNA consistent with an up-regulation of miRNA genes and concomitant down-regulation of miRNA target genes in Pc-G mutants. In addition, although no global effect on DNA methylation and TE activation was revealed in *clf/swn* mutants [15], a subset of TE genes may be regulated by Pc-G proteins (Figure 8 and [51]).

## Discussion

In this study, we analyzed the dynamic regulation of the PRC2-mediated modification H3K27me3 in undifferentiated meristematic and differentiated leaf tissue. Our analyses revealed differential methylation of hundreds of H3K27me3 target genes including protein coding, transposable element and microRNA genes and expand previous observations that plant Pc-G proteins have important roles in controlling tissue-specific expression patterns of gene families and regulatory networks.

### Enrichment of meristematic and leaf tissue for H3K27me3 and expression analyses

To study the dynamics of H3K27me3 in *Arabidopsis* development and its function in tissue differentiation we focused on the vegetative shoot apical meristem which harbors continuously dividing stem cells and gives rise to terminally differentiated organs (leaves) and also differentiates into the flower producing inflorescence meristem. Manual dissection of meristems and young leaves was performed on *clv3* mutants as these have strongly enlarged meristems and increased stem cell numbers [35], but show no obvious defects in leaf development. Independent analyses on dissected wildtype meristems and leaves confirmed differential H3K27me3 of all tested genes (Figure 3). Furthermore, a large overlap of H3K27me3 targets with previously published data on seedlings [5], [20] revealed a similar set of H3K27me3 targets both in wildtype seedlings and *clv3* mutant leaves and meristems. Thus, overall the experimental system appeared highly suitable to study the dynamics of H3K27me3 on a whole genome level during differentiation.

### H3K27me3 is dynamically regulated during differentiation and correlates with gene repression in a tissue-specific manner

Our analyses identified several hundred protein coding genes, transposable elements and microRNA genes exhibiting differential H3K27me3 patterns between meristematic and leaf tissue. Genes that acquired H3K27me3 during differentiation included several meristematic regulators like *STM* or *KNAT2* and *6*, but also many previously non-characterized genes which may present novel regulators of meristem regulation and maintenance. Leaf tissue is developmentally derived from continuously dividing cells in the shoot tip which differentiate in the periphery of the meristem. Thus, H3K27me3 at the subset of genes showing meristem-specific H3K27me3 must be actively or passively removed during differentiation. H3K27me3 demethylases have not been identified in plants yet, however, our analyses strongly suggest the contribution of these enzymatic activities to confer dynamic regulation of H3K27me3. Charron et al [22] observed differential H3K27me3 patterns of protein coding and transposable element genes when dark grown seedlings were compared with dark grown seedlings shifted to light. In addition, dynamic changes in H3K27me3 and/or Pc-G protein binding were also unveiled both during *Drosophila* development and differentiation of mammalian embryonic stem cells suggesting conserved mechanisms which confer developmental dynamics of H3K27me3 [23], [28], [32], [54].

We confirmed that H3K27me3 is largely excluded from heterochomatic regions and transposable element (TE) genes, especially in leaf tissue (only 4% of all targets) [5], [20]. Interestingly, more than 15% of H3K27me3 targets detected in the meristem were TE genes (Figure 2). H3K27me3 detection at TEs was likely not due to cross-reaction of the antibody to H3K27me1 which is found in heterochromatin, because the same anti-H3K27me3 antibody was used for both analyzed tissues. In addition, the antibody was not detecting histones in *clf/swn* mutants which lacked all H3K27me3 but showed no alterations in H3K27me1 (Figure 6, Figure S5). Transposable elements are usually repressed throughout sporophytic development, thus loss of H3K27me3 in leaves likely does not result in transcriptional activation. The stem cells are required for plant germline formation and therefore must be particularly protected from transposable element activation. Thus, H3K27me3 may represent an additional, meristem-specific silencing mechanism besides the canonical heterochromatic marks H3K9me2 and H3K27me1 [55], [56]. Also in mammalian embryonic stem cells, transposable elements are marked by a specific set of repressive histone modifications, suggesting that pluripotent cells are protected by a large array of modifications [32]. For a few TE genes we observed up-regulation in the *clf/swn* Pc-G mutant, consistent with a previous report showing up-regulation of a TE gene in Pc-G mutant endosperm [51], suggesting that Pc-G proteins contribute to the regulation of TEs.

Our data revealed that non-expressed genes are preferential H3K27me3 target genes (Figure 4) as previously reported [5], [20], [21], [31]. Interestingly, protein coding genes which showed strong expression differences between meristem and leaf are enriched for H3K27me3 targets. In addition, differential H3K27me3 was anti-correlated with tissue-specific gene expression patterns (Figure 4). Thus, similar to *Drosophila,* Pc-G proteins likely control ON/OFF expression states of their targets in *Arabidopsis* [30]. However, tissue-specific H3K27me3 is not the only determinant which generates tissue-specific expression patterns as many genes showing differential expression are not covered by H3K27me3.

### Tissue-specific targeting of particular gene families by H3K27me3 in *Arabidopsis*

Previous analyses of H3K27me3 identified transcription factors as one major class of H3K27me3 targets, but preference for certain families was not unveiled [5]. We confirmed this finding as most transcription factor families including *MADS*-, *WOX*-, *HOMEOBOX*- and *YABBY*-transcription factors were preferentially targeted by H3K27me3. However, others including *SPL*-, *ARF*- and *HAP2*-transcription factor genes were largely devoid of H3K27me3, indicating that transcription factor genes are not *per se* enriched for H3K27me3 (see also below). In addition, certain transporter gene families (e.g. sucrose transporters, auxin carriers) and gene families involved in biosynthetic pathways (peroxidases, cytochrome P450 genes) were among the H3K27me3 targets. In *Drosophila* and mammals, transporter and biosynthetic genes (hydrolases, cytochrome P450 genes) were also revealed as preferential H3K27me3 targets in addition to transcription factor genes [6], [23], [26], [29]. This strongly suggests an unexpected level of conservation not only of Pc-G proteins but also of their target gene families between animals and plants.

We also identified gene families and functional categories showing preferential H3K27me3 targeting in the meristem (e.g. *TCP* transcription factor genes, Cytochrome P450 genes; functional categories “plastid” and “kinase activity”) or in leaves (e.g. *MYB* and *HOMEOBOX* transcription factor genes). In particular the differential methylation of *HOMEOBOX* transcription factor genes confirmed the validity of our approach as these are repressed in leaves by Pc-G proteins ([48] and this study).

Thus, our in-depth analyses of H3K27me3 targets confirmed specific gene families as preferential H3K27me3 targets, but also identified novel pathways and gene families that are likely controlled by Pc-G proteins and epigenetic gene regulation.

### Dynamic regulation of miRNA genes by H3K27me3 controls expression of miRNA target genes

Our study discovered a large fraction of microRNA genes as H3K27me3 targets many of which exhibited tissue-specific differences in H3K27me3 (Figure 2, Figure 3, Table 1, Table 2). In addition, we uncovered mis-expression of several miRNA genes and down-regulation of their target genes in Pc-G mutants (Figure 7). Although miRNAs largely post-transcriptionally regulate their target genes, some miRNAs recruit the DNA methylation machinery to their target genes resulting in transcriptional repression [57]–[59]. Similarly to H3K27me3, miRNA genes target a multitude of transcription factor genes [60], [61], raising the possibility that miRNAs mediate recruitment of Pc-G proteins to some of their targets. Our analyses suggest that this is likely a rare phenomenon as transcription factor genes which are miRNA targets, are largely not targeted by H3K27me3 (Table 1, Table 2), despite the general enrichment of transcription factors as H3K27me3 targets ([5] and this study). Nonetheless, we also identified gene pairs, for which both the miRNA gene (e.g. *mir319a*) and their targets (e.g. *TCP2*, *TCP10*) displayed differential, but antagonistic H3K27me3 patterns. Thus, for a subset of genes miRNA-mediated targeting of H3K27me3 is an attractive possibility.

**Figure 9:**
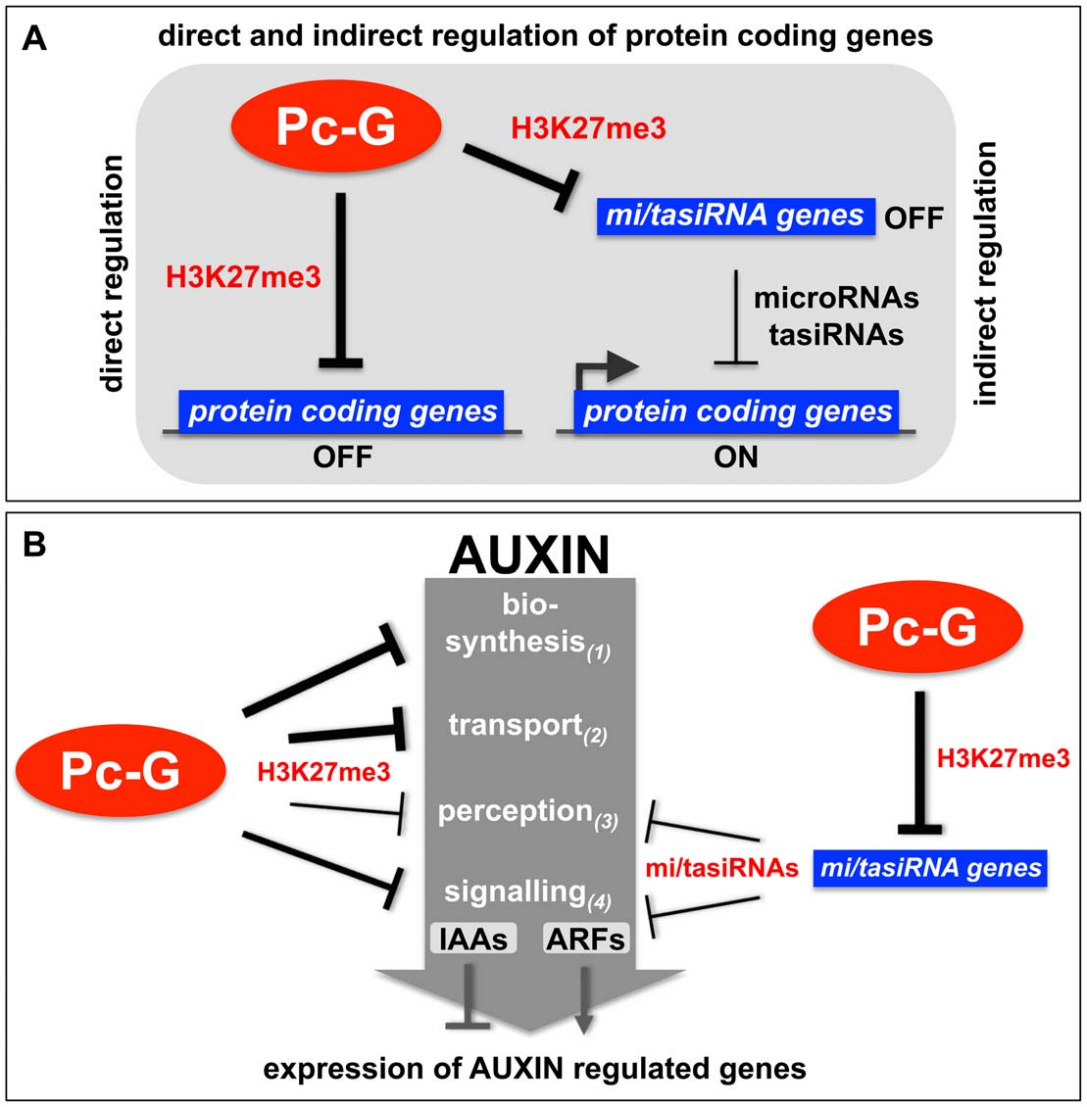
Models of the H3K27me3-mediated regulation of protein coding genes and auxin signaling. (A) Protein coding genes can be directly repressed by Pc-G proteins through the deposition of H3K27me3 or indirectly positively regulated by Pc-G proteins through the H3K27me3-mediated repression of small RNA genes (tasiRNAs and miRNAs) (see Table 1, Table 2 for details). (B) Pc-G proteins directly repress auxin biosynthesis, transport, perception and signaling (*IAA* transcription factors). Thickness of arrows is relative to number of genes targeted by H3K27me3. Pc-G proteins indirectly activate genes involved in auxin perception and signaling (*ARF* transcription factors) through the repression of miRNA (*mir160, 167, 393*) and tasiRNA (*TAS3*) genes. Positive regulation of the *ARF* transcriptional activator and auxin perception genes and negative regulation of *IAA* transcriptional repressor genes likely promotes expression of auxin inducible genes (see Table 3, Table 4 for details).

In summary, our studies provide evidence that Pc-G proteins and H3K27me3 do not only negatively regulate expression of transcription factor genes by direct association, but also positively control their expression by restricting expression of miRNA genes (Figure 9). This regulation appears to be particularly important in meristematic and leaf tissues as a large fraction of miRNA genes is differentially methylated in these two tissues (Figure 2). Regulation of miRNA genes by Pc-G proteins is likely a conserved mechanism. Recent analyses of miRNA gene regulation during mouse and human lymphopoiesis and genome-wide profiling of Pc-G protein binding sites in *Drosophila* uncovered H3K27me3-mediated control of miRNA gene expression [62], [63]. In addition, more than 30 miRNA loci were discovered as H3K27me3 targets in human embryonic stem cells [26].

### H3K27me3 regulates diverse pathways in auxin biosynthesis, transport, perception, and signaling

Organ-specific auxin maxima are generated by both local auxin biosynthesis and auxin transport, which is largely mediated by the PIN efflux carrier proteins (reviewed in [43], [64], [65]). To date, three different pathways for the synthesis of auxin from tryptophan have been identified or proposed for plants (reviewed in [42]). Our analyses identified most of the key regulators of auxin transport and biosynthesis to be H3K27me3 targets several of which are leaf-specifically methylated (Figure 3, Figure 5, Table 3, Table 4). Additionally, our results suggest that auxin perception is controlled by Pc-G proteins as H3K27me3 targets the *mir393a* gene which in turn regulates the auxin receptor genes *TIR1/AFB* (Table 4) [66], [67]. In addition, H3K27me3 regulates the transcriptional responses to auxin as half of the *AUX/IAA* repressor genes are covered by H3K27me3. Although only one of the 23 *ARF* transcriptional activator genes carries H3K27me3, all known miRNAs or tasiRNAs that regulate a total of 8 *ARF* genes are H3K27me3 targets (Table 2). Thus, we propose that auxin responsive genes are largely suppressed in Pc-G mutants as H3K27me3 restricts expression of the *AUX/IAA* repressors and promotes expression of the *ARF* activators by controlling the expression of *ARF*-regulating miRNAs (Figure 7, Figure 9, Table 2). Consistent with this hypothesis, we observed down-regulation of several auxin-inducible *PIN* genes (*PIN3*, *PIN4*, *PIN7*) [68] in *clf/swn* mutants (Figure S7). In addition, the Pc-G mutant *terminal flower2* was recently shown to have lower expression of auxin responsive genes including the synthetic reporter DR5::GUS [69] and genome-wide expression profiles of *embryonic flower 1 (emf1)* and *emf2* Pc-G mutants identified more down- than upregulated auxin regulatory genes in the mutants [70]. Loss of vegetative Pc-G activity in *clf/swn* or *vrn2/emf2* mutants is accompanied by loss of organ identity, generation of somatic embryos and callus-like appearance [18], [19] and all these phenotypes are associated with deregulated auxin responses. Although we cannot rule out that these phenotypes are auxin-independent, we showed mis-regulation of *PIN, ARF* and *miRNA167* genes in Pc-G mutants revealing that loss of H3K27me3 is indeed associated with mis-regulation of genes involved in auxin responses (Figure 7). Other studies have also linked Pc-G proteins to hormonal control of gene expression: Charron and colleagues [22] uncovered strong H3K27me3 targeting of genes involved in gibberellic acid biosynthesis and inactivation. Global gene expression in *emf1* and *emf2* mutants revealed mis-regulation of many genes involved in diverse hormonal pathways [70]. However, many of these effects are likely indirect, thus a rigorous comparison of Pc-G protein binding, H3K27me3 targeting and gene expression profiles in Pc-G mutants in defined tissues will be essential to uncover the mis-regulated target genes that determine the Pc-G mutant phenotypes.

### Role of H3K27me3 in Pc-G silencing and epigenetic inheritance

This and previous studies uncovered at least 25% of all *Arabidopsis* protein coding genes as H3K27me3 targets, some of which are tissue-specifically methylated [5], [20]–[22], [31]. Although H3K27me3 is entirely dependent on Pc-G proteins, H3K4me3 is enriched in *clf/swn* mutants (Figure 6) and H3K27me3 is strongly correlated with gene repression (Figure 4), only a limited number of H3K27me3 targets are mis-expressed in Pc-G mutants or in tissues in which H3K27me3 is lost (this study and [70]). Also in animals lack of Pc-G proteins results in mis-expression of only a small number of target genes [24]. Thus, loss of H3K27me3 may poise genes for activation but gene expression may require additional environmental or developmental cues. Thus, Pc-G proteins and H3K27me3 may confer stability of developmental processes and gene regulatory networks.

Pc-G proteins are considered as epigenetic regulators conferring mitotically stable gene expression states. The large number of H3K27me3 targets implies that either more than 25% of all protein coding genes are epigenetically regulated or that H3K27me3 has a more general gene regulatory role. Certainly, epigenetic phenomena like the vernalization response are controlled by Pc-G proteins and H3K27me3 [8], [71], [72]. In addition, Pc-G target genes are covered with H3K27me3 which may ensure perpetuation of the modification through replication [9]. Interestingly, for small genes like miRNA genes the number of modified nucleosomes may not be sufficient to guarantee high fidelity of inheritance.

This and previous studies unveiled tissue-specific dynamics of H3K27me3 distribution [22], [31], [51], suggesting that if H3K27me3 is generally mitotically heritable it may persist for only limited cell divisions until it is reset again to allow gene activation. The mechanisms of H3K27me3 inheritance and resetting can now be studied with the identified Pc-G target genes which are dynamically regulated during differentiation.

## Materials and Methods

### Plant material and growth conditions

All plants were grown under either long day (16 h light/8 h darkness) or short day conditions (8 h light/16 h darkness). The *clavata3*–*9* mutant in Columbia background carries a point mutation in the *CLV3* coding region which disrupts *CLV3* function. *clf-28* (SALK_139371), *swn-7* (SALK_109121), *clf-28 swn-7*, *vrn2-1* [47], *emf2-10* [18] and *vrn2-1/emf2*–*10* mutants were used in this study. *Pmir167a::GUS*, *Pmir167d::GUS* and *pARF6::ARF6-GUS* were previously described [53]. For expression and ChIP-chip analyses, *clv3* mutant plants were grown for 9 weeks under short day conditions and enriched for meristematic and leaf tissue by manual dissection. Tissues for expression and ChIP-chip analyses were simultaneously harvested. For zebularine treatment, plants were germinated on plates containing 40 µM zebularine (Sigma, Munich) and harvested 9 days after germination.

### Array-based expression analysis of different tissue samples

#### Array hybridization

Total RNA was extracted with QIAGEN plant RNeasy kit (Qiagen, Hilden), Superscript II (Invitrogen, Carlsbad) was used for cDNA synthesis. Probe labelling and array hybridisation on Agilent 44 k arrays [G2519F, V4 (Agilent, Santa Clara)] were performed by the Imagenes Corp. (Berlin, Germany).

#### Gene expression data analysis

The quality of the array hybridization was tested with scatter plots of the raw intensities (Figure S9). Pearson correlation coefficients were determined showing high similarity of biological replicates. The normalized and background subtracted data received from Imagenes was processed with a spreadsheet program (Excel, Microsoft) resulting in 22988 expressed genes (total), 22237 in the meristem (Me) and 22017 in the leaf (Le) sample (Figure S2). For the comparison of different tissues the log2 values [log2(Me); log2(Le)] of the normalized intensities and their difference between the tissues [Δlog2(Me-Le)] were calculated. Gene sets of different expression levels were binned to compare gene expression and histone methylation (Figure 4). Genes with a minimal difference in expression of Δlog2|Me-Le|≥2 which were statistically significantly different [determined by Students tTest (*p*≤0.05)] were considered as tissue specifically expressed. Genes with Δlog2|Me-Le| <1 and a standard deviation of the samples ≤0.05% were considered as equally expressed (Figure S2). Genes expressed in the different tissues area are provided in Table S4.

### Tissue-specific H3K27me3 ChIP and ChIP-chip analyses

#### ChIP and array hybridization

For each tissue, 200 mg plant material was harvested on ice, crosslinked with 1% formaldehyde for 15 minutes under vacuum, frozen in liquid nitrogen and stored at −70° until chromatin extraction. A previously described protocol [8] was used for ChIP. Antibodies specific for H3K27me3 (#07-449) and H3K4me3 (#07-473) were retrieved from Millipore (Billerica, USA). Chromatin was sheared with a Bioruptor sonicator (10 pulses, 30 seconds each) (Diagenode, Liege, Belgium). High density tiling arrays with probes tiled approximately every 220 bp over the entire sequenced *Arabidopsis* genome were used as described [36], [37]. Input and precipitated DNA was amplified with a whole genome amplification kit (WGA2, Sigma, St. Louis) as described in “PROT30” of The Epigenome NoE web-page (http://www.epigenome-noe.net/). Labelling and array hybridisation were performed by the Imagenes NimbleGen service (Berlin, Germany).

#### H3K27me3 ChIP-chip data analysis

The array hybridization data was received from Imagenes (Berlin, Germany) as intensity ratios of precipitate and input samples in the GFF-file format. In a first processing step M-Values [M = log2(IP/I) where IP is signal intensity from hybridized immunoprecipitated DNA and I is signal intensity from hybridized input DNA] were determined. The quality of the array hybridization was analyzed with scatter plots of the M values for biological replicates (Figure S10). The correlation of the two biological replicates for each tissue was estimated with the Spearman's rank correlation coefficient (SCC) (Figure S10). The data was analyzed with SignalMap1.9 software (Nimblegen) and the Bioconductor R packages [73] in an R programming environment. M values were used as input for the Bioconductor package *Ringo*. The *Ringo* clustering algorithm has been developed specifically for widely occurring enrichments including those found for histone modifications [74]. Prior to cluster detection *Ringo* applies a running median to smooth the datasets. A window size of 1400 bp was used for running median. The cluster algorithm was executed with a distance cut off of 700 bp and an intensity threshold defined by the 80th percentile. The identified methylation clusters were used to calculate gene specific methylation scores for each annotation [based on TAIR8 genome annotation (www.arabidopsis.org)] and tissue (Me or Le) and scaled so that the maximum equals 100. Further analyses and comparisons of loci-specific methylation scores were calculated with a regular spreadsheet program (Excel, Microsoft). A scale normalisation on the level of methylation scores was performed for the different tissues to account for differences in precipitation efficiency. Since the array hybridisations were performed as replicates we only considered genes for which a positive methylation score was detected in both samples. This resulted in 6071 protein coding genes methylated in the meristematic sample and 6928 in leaf sample (Figure S11). Other annotations are depicted in Table S1. We grouped methylated genes in specific classes for further analyses after retrieving methylation scores (ms) for each annotation: methylated [ms (Me)>0 (Me>0 in the text) or ms(Le)>0 (Le>0) (target)], non methylated [ms(Me) = 0 and ms(Le) = 0 (Me = Le = 0) (non target)], equally [ms(Me) = ms(Le)>0 (M = L)] or differentially methylated in the meristem (ms(Me)≫ms(Le) [dM+]) or leaf sample (ms(Le)≫ms(Me) [dL+]). Two criteria were used to detect differentially methylated loci: ms(Me)/ms(Le)≥2 or ms(Le) = 0 (*p*≤0.05, Students tTest) and |ms(Me)-ms(Le)|≥10 for meristem specifically methylated loci (dM+) and ms(Le)/ms(Me)≥2 or ms(Me) = 0 (*p*≤0.05, Student’s tTest) and |ms(Me)-ms(Le)|≥10 for leaf specifically methylated loci (dL+). Equally methylated loci (M = L) were isolated with the criteria 0.5<ms(Me)/ms(Le)<2; |ms(Me)-ms(Le)|<10 and a standard deviation ≤0.05%. H3K27me3 target genes identified in the different tissues are listed in Table S5.

### Gene Ontology (GO) term and gene family analyses of target genes

Specific gene ontologies (GO) were extracted for specific gene sets with the GO analysis tool on TAIR (www.arabidopsis.org/tools/bulk//go/index.jsp) and calculated as enrichment or depletion over the background of all protein coding genes in the genome (based on TAIR8). Significance was determined by a hypergeometric test. Gene families were extracted from TAIR and the plant transcription factor database (http://plntfdb.bio.uni-potsdam.de/v3.0/) [75] and compared to specific subsets of methylated genes. Significance of over- or under-representation of target genes within a gene family was tested with a χ^2^-test (in comparison with the observed frequency of targets or subsets within the genome).

### qPCR based analysis of gene expression (qRT-PCR) and histone modifications (ChIP-qPCR)

All qPCR analyses were performed with a CHROMO4 cycler (BioRad, Munich) and a MesaGreen reaction mix (Eurogentec, Liege) with a two step program. The genes At5g60390 (EF-1α), At4g34270 and At1g13320 were used as reference genes in qRT-PCR based expression analyses [76]. For ChIP-qPCR analyses the PCRs were performed on input and immunoprecipitated samples, % of input was calculated and normalized to the reference gene *FUSCA3* (*FUS3*, AT3G26790) which carries H3K27me3 and is not expressed in both meristematic and leaf sample. For H3K4me3 analyses, *ACTIN2* (AT5G09810) was used as reference as it carries H3K4me3 and is equally expressed in both samples. Sequences of oligonucleotides used for gene expression and ChIP analyses are listed in Table S6.

### Analyses of global histone modification in Pc-G mutants by immunoblot analyses

Histone enriched plant extracts were prepared from ten day old seedlings as described [77]. Various histone modifications were detected by standard immunoblot analyses using antibodies specific for H3 (Abcam; ab1791), H3K27me1 (Millipore; 07-448), H3K27me2 (Millipore; 07-452), H3K27me2 (Active Motif; 39245), H3K27me3 (Millipore; 07-449), H3K4me3 (Diagenode; pAb-003-050).

### Histochemical analysis of *Arabidopsis* plants

Seven days old plants were histochemically assayed as previously described [78]. Photos were taken with a stereomicroscope equipped with AxioCam ICC1 camera and AxioVision4.8 software (Zeiss).

### Accession numbers

Data files for transcriptome and H3K27me3 analyses can be accessed via GEO accession number GSE24507.

## Supporting Information

Figure S1 Tissue-specific differences of H3K4me3 and H3K27me3. (A) Comparison of the H3K27me3 profiles derived from meristem (Me) and leaf (Le) tissue reveal differences in the H3K27me3 abundance within the *KNAT6* locus. Genes are depicted in blue, the signal intensity ratio (IP/input) of each array probe in green, the position of the qPCR-probes (KNAT6 pA, pK1, pK2) in red. (B, C) ChIP with H3K27me3 (B) and H3K4me3 (C) specific antibodies followed by qPCR analysis of the precipitates. Plant material was dissected from 9 weeks old, short day grown *clv3* mutants. For the tissue comparison the data was normalized and is presented as enrichment to the corresponding reference loci “*KNAT6 pK2*” (AT1G23320) (for H3K27me3 ChIP) and “*ACT pATG*” (AT5G09810) (for H3K4me3 ChIP). Region pK1 (at1g23360) does not show enrichment in H3K27me3 in array (A) and ChIP-qPCR (B).
(TIF)

Figure S2 Identification of equally and differentially expressed genes. The diagram shows the number of expressed genes identified in meristem (Me) and leaf (Le) tissues as well as the intersection of both tissues (upper panel) (means of two biological replicates). Genes harbouring equal or differential expression levels were identified as described in methods (lower panel).
(TIF)

Figure S3 Functional categorization of H3K27me3 target genes by GO term analysis. (A–C) Frequency of H3K27me3 target (blue), non target (black), equally (grey), meristem- (yellow) and leaf-specifically (green) methylated genes within functional annotations (GO terms) relative to all protein coding genes of the TAIR8 *Arabidopsis* genome annotation. The analysis is split in the GO_slim analyses of cellular components (A), of molecular functions (B) and biological processes (C). Significant differences (hypergeometric test) are only given for dM+, dL+, Differences for target and non-target genes were all significant (p<0.001).
(TIF)

Figure S4 Abundance of H3K27me3 target genes in gene families. (A, C, E) Relative enrichment or depletion of H3K27me3 target genes (blue) or equally methylated genes (M = L, purple) in specific transporter (A), regulatory RNA (C), enzyme and other families (E). (B, D, F) Relative enrichment or depletion of meristem-specific (dM+, yellow) or leaf-specific (dM+, green) H3K27me3 target genes in specific transporter (B), small RNA (D), enzyme and other families (F). Y-axes indicate fold difference relative to the observed frequencies of specific subsets within the genome. Asterisks indicate significant differences (*p*≤0.05, χ^2^-test) compared to total number of genes for a specific subset (adjacent subsets with same significance are labelled with coloured brackets). Details of statistical analyses can be found in Table S3. Gene family names are shown in G. (H) Relative enrichment or depletion of H3K27me3 target genes in retrotransposon and DNA transposon genes. Retrotransposon and DNA transposon genes were grouped. (I) Relative enrichment or depletion of H3K27me3 target genes in subclasses of retrotransposon and DNA transposon genes (based on TAIR8 genome annotation). Y-axes indicate fold difference relative to the observed frequencies of specific subsets within the genome. Asterisks indicate significant differences (*p*≤0.05, χ^2^-test) compared to total number of genes for a specific subset. Details of statistical analyses can be found in Table S3.
(TIF)

Figure S5 Immunoblot analyses of H3K27me1 and H3K27me2 in Pc-G mutants. Immunoblot analyses of H3K27me1 and H3K27me2 levels in wildtype and Pc-G mutants. Similar amounts of histone H3 was loaded as confirmed with a H3 specific antibody detecting the unmodified C-terminal part of H3. Same plant material was used as in Figure 6.
(TIF)

Figure S6 Gene-specific analyses of H3K27me3 in wildtype and *vrn2-1*/*emf2-10* leaves. H3K27me3 target genes were analyzed in leaf material derived from 70d old, short day grown wild type and *vrn2-1*/*emf2-10* plants by ChIP-qPCR. The qPCR data is presented as enrichment to the reference locus *FUS3* (AT3G26790) as described in Figure 3, enrichment factors wt compared to *vrn2-1/emf2-10* are indicated in brackets. The results are shown as mean values of two biological replicates.
(TIF)

Figure S7 Regulation of *PIN* gene expression in Pc-G mutants. (A)–(B) qRT-PCR based expression analyses of (A) 9 d old wildtype and *clf-28*/*swn-7* plants or (B) 45 days old leaves of wildtype and *vrn2-1*/*emf2-10* plants. Expression values were normalized to At5g60390 (EF-1α).
(TIF)

Figure S8 Colorimetric analyses of *pMIR167d::GUS* in wildtype and *clf/swn* mutants. Colorimetric analyses of *pMir167d::GUS* expression in 9 d old wildtype (A) and *clf-28/swn-7* double mutants (F). Insets are close-ups. Asterisks mark meristematic region, white arrow base of hypocotyls. Scale bars indicate 1 cm. All plants were grown in short day conditions.
(TIF)

Figure S9 Comparison of expression arrays. Microarray hybridization intensities (XM1, XM2  =  meristem expression profiles; XL1, XL2  = leaf expression profiles) were compared in a matrix of scatter plots revealing high overlap for biological replicates. The density of data points is represented by a darker colouration. White areas correspond to areas without data points. In low density areas single data points are indicated by single dots. The Pearson correlation coefficient (PCC) is shown below the diagonal.
(TIF)

Figure S10 Correlation of H3K27me3 arrays (ChIP-chip) on the level of raw intensity values. The matrix plot correlates H3K27me3 ChIP-chip signal intensities between samples. L1 and L2 are biological replicates of leaf tissue, M1 and M2 of meristematic tissue. The upper triangle of the matrix displays scatter plots to compare each sample to the other samples. The axes represent the M-Values (M = log2(R)-log2(G) with R(red) = IP and G(green) = input) for all probes of the according sample. The intensity of shading of the scatter plot represents the density of data points within that area. Where density of data points is low single data points are shown. The lower triangle shows Spearman's rank correlation coefficients between different samples revealing high overlap between biological replicates.
(TIF)

Figure S11 Comparison of identified H3K27me3 target genes in biological replicates. The diagram shows the identified numbers of H3K27me3 target genes for biological replicates for meristem (Me) or leaf (Le) material. The arrays of the same tissue share 85.2% (Me1 and Me2) and 89.9% (Le1 and Le2), respectively (two upper panels). Target genes identified in both replicates were used for the tissue comparison (lower panel and Figure 2).
(TIF)

Table S1 Summary of all identified H3K27me3 target gene loci. All annotations of TAIR8 (www.arabidopsis.org) were analysed for their H3K27me3 coverage in leaf and meristematic tissue. The table shows the total numbers of identified target loci in comparison to the analysis of Zhang et al. [5] and Oh et al. [20]. TEs: transposable elements, ncRNAs: non coding RNAs. See methods for details on different subsets of meristem and leaf-specific H3K27me3 targets.
(TIF)

Table S2 Analysis of the significance of over- or under-representation of target genes within expression gene sets. Comparisons were performed between the frequency of a given H3K27me3 subset and the frequency of expression set in the genome (upper table) or between the frequency of a given expression set and the frequency of H3K27me3 subset in the genome (lower table). X^2^-test was applied to identify significantly different genes (p≤0.05).
(TIF)

Table S3 Frequency of H3K27me3 target genes within gene families. The tables (A–E) show the percentages of H3K27me3 target genes, equally (M = L), meristem (dM+) and leaf (dL+) specifically methylated genes for transcription factor (A), transporter (B), other gene families (C, D) and transposable element genes (E). The probabilities that the observed frequencies within a gene family match the average representation in the genome [*p*(ga)] are given by a X^2^-test. Frequencies resulting in p≤0.05 were considered as significantly different. For transcription factor genes, the probabilities that the observed frequencies match the average of all transcription factor genes [*p*(TF)] were also calculated.
(TIF)

Table S4 Lists of equally and differentially expressed genes. Genes harbouring specific expression levels are grouped and shown as gene sets X(M = L), dXM≥2 (Δlog2(M-L)≥2) and dXL≤−2 (Δlog2(M-L)≤−2).
(XLSX)

Table S5 Lists of H3K27me3 target genes identified by ChIP-chip. Enriched genes were separately identified for each tissue (Me, Le) and methylation scores were calculated for each gene as described in methods. All genes harbouring H3K27me3 scores >0 are included in the lists. Genes harbouring defined methylation levels were identified as described above (Figure 2 and methods).
(XLSX)

Table S6 Oligonucleotides used for ChIP-qPCR and qRT-PCR analyses.
(XLSX)

## References

[1] Barton MK (2010). Twenty years on: the inner workings of the shoot apical meristem, a developmental dynamo.. Dev Biol.

[2] Schuettengruber B, Chourrout D, Vervoort M, Leblanc B, Cavalli G (2007). Genome regulation by polycomb and trithorax proteins.. Cell.

[3] Muller J, Verrijzer P (2009). Biochemical mechanisms of gene regulation by polycomb group protein complexes.. Curr Opin Genet Dev.

[4] Johnson L, Mollah S, Garcia BA, Muratore TL, Shabanowitz J (2004). Mass spectrometry analysis of Arabidopsis histone H3 reveals distinct combinations of post-translational modifications.. Nucleic Acids Res.

[5] Zhang X, Clarenz O, Cokus S, Bernatavichute YV, Pellegrini M (2007). Whole-genome analysis of histone H3 lysine 27 trimethylation in Arabidopsis.. PLoS Biol.

[6] Schwartz YB, Kahn TG, Nix DA, Li XY, Bourgon R (2006). Genome-wide analysis of Polycomb targets in Drosophila melanogaster.. Nat Genet.

[7] Bernstein BE, Mikkelsen TS, Xie X, Kamal M, Huebert DJ (2006). A bivalent chromatin structure marks key developmental genes in embryonic stem cells.. Cell.

[8] Schubert D, Primavesi L, Bishopp A, Roberts G, Doonan J (2006). Silencing by plant Polycomb-group genes requires dispersed trimethylation of histone H3 at lysine 27.. EMBO J.

[9] Hansen KH, Bracken AP, Pasini D, Dietrich N, Gehani SS (2008). A model for transmission of the H3K27me3 epigenetic mark.. Nat Cell Biol.

[10] Muller J, Hart CM, Francis NJ, Vargas ML, Sengupta A (2002). Histone methyltransferase activity of a Drosophila Polycomb group repressor complex.. Cell.

[11] Cao R, Wang L, Wang H, Xia L, Erdjument-Bromage H (2002). Role of histone H3 lysine 27 methylation in Polycomb-group silencing.. Science.

[12] Czermin B, Melfi R, McCabe D, Seitz V, Imhof A (2002). Drosophila enhancer of Zeste/ESC complexes have a histone H3 methyltransferase activity that marks chromosomal Polycomb sites.. Cell.

[13] Kuzmichev A, Nishioka K, Erdjument-Bromage H, Tempst P, Reinberg D (2002). Histone methyltransferase activity associated with a human multiprotein complex containing the Enhancer of Zeste protein.. Genes Dev.

[14] Jiang D, Wang Y, Wang Y, He Y (2008). Repression of FLOWERING LOCUS C and FLOWERING LOCUS T by the Arabidopsis Polycomb repressive complex 2 components.. PLoS ONE.

[15] Lindroth AM, Shultis D, Jasencakova Z, Fuchs J, Johnson L (2004). Dual histone H3 methylation marks at lysines 9 and 27 required for interaction with CHROMOMETHYLASE3.. EMBO J.

[16] Schatlowski N, Creasey K, Goodrich J, Schubert D (2008). Keeping plants in shape: Polycomb-group genes and histone methylation.. Semin Cell Dev Biol.

[17] Kohler C, Villar CB (2008). Programming of gene expression by Polycomb group proteins.. Trends Cell Biol.

[18] Chanvivattana Y, Bishopp A, Schubert D, Stock C, Moon YH (2004). Interaction of Polycomb-group proteins controlling flowering in Arabidopsis.. Development.

[19] Schubert D, Clarenz O, Goodrich J (2005). Epigenetic control of plant development by Polycomb-group proteins.. Curr Opin Plant Biol.

[20] Oh S, Park S, van NS (2008). Genic and global functions for Paf1C in chromatin modification and gene expression in Arabidopsis.. PLoS Genet.

[21] Turck F, Roudier F, Farrona S, Martin-Magniette ML, Guillaume E (2007). Arabidopsis TFL2/LHP1 specifically associates with genes marked by trimethylation of histone H3 lysine 27.. PLoS Genet.

[22] Charron JB, He H, Elling AA, Deng XW (2009). Dynamic landscapes of four histone modifications during deetiolation in Arabidopsis.. Plant Cell.

[23] Schuettengruber B, Ganapathi M, Leblanc B, Portoso M, Jaschek R (2009). Functional anatomy of polycomb and trithorax chromatin landscapes in Drosophila embryos.. PLoS Biol.

[24] Bracken AP, Dietrich N, Pasini D, Hansen KH, Helin K (2006). Genome-wide mapping of Polycomb target genes unravels their roles in cell fate transitions.. Genes Dev.

[25] Boyer LA, Plath K, Zeitlinger J, Brambrink T, Medeiros LA (2006). Polycomb complexes repress developmental regulators in murine embryonic stem cells.. Nature.

[26] Lee TI, Jenner RG, Boyer LA, Guenther MG, Levine SS (2006). Control of developmental regulators by Polycomb in human embryonic stem cells.. Cell.

[27] Squazzo SL, O'Geen H, Komashko VM, Krig SR, Jin VX (2006). Suz12 binds to silenced regions of the genome in a cell-type-specific manner.. Genome Res.

[28] Oktaba K, Gutierrez L, Gagneur J, Girardot C, Sengupta AK (2008). Dynamic regulation by polycomb group protein complexes controls pattern formation and the cell cycle in Drosophila.. Dev Cell.

[29] Tolhuis B, de Wit E, Muijrers I, Teunissen H, Talhout W (2006). Genome-wide profiling of PRC1 and PRC2 Polycomb chromatin binding in Drosophila melanogaster.. Nat Genet.

[30] Papp B, Muller J (2006). Histone trimethylation and the maintenance of transcriptional ON and OFF states by trxG and PcG proteins.. Genes Dev.

[31] Deal RB, Henikoff S (2010). A Simple Method for Gene Expression and Chromatin Profiling of Individual Cell Types within a Tissue.. Developmental Cell.

[32] Mikkelsen TS, Ku M, Jaffe DB, Issac B, Lieberman E (2007). Genome-wide maps of chromatin state in pluripotent and lineage-committed cells.. Nature.

[33] Azuara V, Perry P, Sauer S, Spivakov M, Jorgensen HF (2006). Chromatin signatures of pluripotent cell lines.. Nat Cell Biol.

[34] Lafos M, Schubert D (2009). Balance of power—dynamic regulation of chromatin in plant development.. Biol Chem.

[35] Fletcher JC, Brand U, Running MP, Simon R, Meyerowitz EM (1999). Signaling of cell fate decisions by CLAVATA3 in Arabidopsis shoot meristems.. Science.

[36] Zilberman D, Gehring M, Tran RK, Ballinger T, Henikoff S (2007). Genome-wide analysis of Arabidopsis thaliana DNA methylation uncovers an interdependence between methylation and transcription.. Nat Genet.

[37] Thibaud-Nissen F, Wu H, Richmond T, Redman JC, Johnson C (2006). Development of Arabidopsis whole-genome microarrays and their application to the discovery of binding sites for the TGA2 transcription factor in salicylic acid-treated plants.. Plant J.

[38] Semiarti E, Ueno Y, Tsukaya H, Iwakawa H, Machida C (2001). The ASYMMETRIC LEAVES2 gene of Arabidopsis thaliana regulates formation of a symmetric lamina, establishment of venation and repression of meristem-related homeobox genes in leaves.. Development.

[39] Fitzgerald JN, Hui PS, Berger F (2009). Polycomb group-dependent imprinting of the actin regulator AtFH5 regulates morphogenesis in Arabidopsis thaliana.. Development.

[40] Gustafson AM, Allen E, Givan S, Smith D, Carrington JC (2005). ASRP: the Arabidopsis Small RNA Project Database.. Nucleic Acids Res.

[41] Chandler JW (2009). Local auxin production: a small contribution to a big field.. Bioessays.

[42] Zhao Y (2010). Auxin biosynthesis and its role in plant development.. Annu Rev Plant Biol.

[43] Vanneste S, Friml J (2009). Auxin: a trigger for change in plant development.. Cell.

[44] Liscum E, Reed JW (2002). Genetics of Aux/IAA and ARF action in plant growth and development.. Plant Mol Biol.

[45] Sung S, Schmitz RJ, Amasino RM (2006). A PHD finger protein involved in both the vernalization and photoperiod pathways in Arabidopsis.. Genes Dev.

[46] Calonje M, Sanchez R, Chen L, Sung ZR (2008). EMBRYONIC FLOWER1 participates in polycomb group-mediated AG gene silencing in Arabidopsis.. Plant Cell.

[47] Gendall AR, Levy YY, Wilson A, Dean C (2001). The VERNALIZATION 2 gene mediates the epigenetic regulation of vernalization in Arabidopsis.. Cell.

[48] Katz A, Oliva M, Mosquna A, Hakim O, Ohad N (2004). FIE and CURLY LEAF polycomb proteins interact in the regulation of homeobox gene expression during sporophyte development.. Plant J.

[49] Xu L, Shen WH (2008). Polycomb silencing of KNOX genes confines shoot stem cell niches in Arabidopsis.. Curr Biol.

[50] Palatnik JF, Allen E, Wu X, Schommer C, Schwab R (2003). Control of leaf morphogenesis by microRNAs.. Nature.

[51] Weinhofer I, Hehenberger E, Roszak P, Hennig L, Kohler C (2010). H3K27me3 profiling of the endosperm implies exclusion of polycomb group protein targeting by DNA methylation.. PLoS Genet.

[52] Baubec T, Pecinka A, Rozhon W, Mittelsten SO (2009). Effective, homogeneous and transient interference with cytosine methylation in plant genomic DNA by zebularine.. Plant J.

[53] Wu MF, Tian Q, Reed JW (2006). Arabidopsis microRNA167 controls patterns of ARF6 and ARF8 expression, and regulates both female and male reproduction.. Development.

[54] Negre N, Hennetin J, Sun LV, Lavrov S, Bellis M (2006). Chromosomal distribution of PcG proteins during Drosophila development.. PLoS Biol.

[55] Jackson JP, Johnson L, Jasencakova Z, Zhang X, PerezBurgos L (2004). Dimethylation of histone H3 lysine 9 is a critical mark for DNA methylation and gene silencing in Arabidopsis thaliana.. Chromosoma.

[56] Jacob Y, Feng S, LeBlanc CA, Bernatavichute YV, Stroud H (2009). ATXR5 and ATXR6 are H3K27 monomethyltransferases required for chromatin structure and gene silencing.. Nat Struct Mol Biol.

[57] Grigg SP, Canales C, Hay A, Tsiantis M (2005). SERRATE coordinates shoot meristem function and leaf axial patterning in Arabidopsis.. Nature.

[58] Chellappan P, Xia J, Zhou X, Gao S, Zhang X (2010). siRNAs from miRNA sites mediate DNA methylation of target genes.. Nucleic Acids Res.

[59] Bao N, Lye KW, Barton MK (2004). MicroRNA binding sites in Arabidopsis class III HD-ZIP mRNAs are required for methylation of the template chromosome.. Dev Cell.

[60] Jones-Rhoades MW, Bartel DP (2004). Computational identification of plant microRNAs and their targets, including a stress-induced miRNA.. Mol Cell.

[61] Rhoades MW, Reinhart BJ, Lim LP, Burge CB, Bartel B (2002). Prediction of plant microRNA targets.. Cell.

[62] Kuchen S, Resch W, Yamane A, Kuo N, Li Z (2010). Regulation of microRNA expression and abundance during lymphopoiesis.. Immunity.

[63] Enderle D, Beisel C, Stadler MB, Gerstung M, Athri P (2011). Polycomb preferentially targets stalled promoters of coding and noncoding transcripts.. Genome Res.

[64] Zhao Y (2008). The role of local biosynthesis of auxin and cytokinin in plant development.. Curr Opin Plant Biol.

[65] Zazimalova E, Murphy AS, Yang H, Hoyerova K, Hosek P (2010). Auxin transporters—why so many?. Cold Spring Harb Perspect Biol.

[66] Parry G, Calderon-Villalobos LI, Prigge M, Peret B, Dharmasiri S (2009). Complex regulation of the TIR1/AFB family of auxin receptors.. Proc Natl Acad Sci U S A.

[67] Navarro L, Dunoyer P, Jay F, Arnold B, Dharmasiri N (2006). A plant miRNA contributes to antibacterial resistance by repressing auxin signaling.. Science.

[68] Goda H, Sasaki E, Akiyama K, Maruyama-Nakashita A, Nakabayashi K (2008). The AtGenExpress hormone and chemical treatment data set: experimental design, data evaluation, model data analysis and data access.. Plant J.

[69] Rizzardi K, Landberg K, Nilsson L, Ljung K, Sundås-Larsson A (2010). TFL2/LHP1 is involved in auxin biosynthesis through positive regulation of YUCCA genes.. Plant J.

[70] Kim SY, Zhu T, Sung ZR (2010). Epigenetic regulation of gene programs by EMF1 and EMF2 in Arabidopsis.. Plant Physiol.

[71] Sung S, Amasino RM (2004). Vernalization in Arabidopsis thaliana is mediated by the PHD finger protein VIN3.. Nature.

[72] Bastow R, Mylne JS, Lister C, Lippman Z, Martienssen RA (2004). Vernalization requires epigenetic silencing of FLC by histone methylation.. Nature.

[73] Gentleman RC, Carey VJ, Bates DM, Bolstad B, Dettling M (2004). Bioconductor: open software development for computational biology and bioinformatics.. Genome Biol.

[74] Toedling J, Skylar O, Krueger T, Fischer JJ, Sperling S (2007). Ringo—an R/Bioconductor package for analyzing ChIP-chip readouts.. BMC Bioinformatics.

[75] Perez-Rodriguez P, Riano-Pachon DM, Correa LG, Rensing SA, Kersten B (2010). PlnTFDB: updated content and new features of the plant transcription factor database.. Nucleic Acids Res.

[76] Czechowski T, Stitt M, Altmann T, Udvardi MK, Scheible WR (2005). Genome-wide identification and testing of superior reference genes for transcript normalization in Arabidopsis.. Plant Physiol.

[77] Yan D, Zhang Y, Niu L, Yuan Y, Cao X (2007). Identification and characterization of two closely related histone H4 arginine 3 methyltransferases in Arabidopsis thaliana.. Biochem J.

[78] Colon-Carmona A, You R, Haimovitch-Gal T, Doerner P (1999). Technical advance: spatio-temporal analysis of mitotic activity with a labile cyclin-GUS fusion protein.. Plant J.

