# Supplementary figures and images for: Dynamic Regulation of H3K27 Trimethylation during Arabidopsis Differentiation
Source: PLoS Genet. 2011 Apr 7;7(4):e1002040. doi: 10.1371/journal.pgen.1002040 (PMC3072373; doi:10.1371/journal.pgen.1002040)

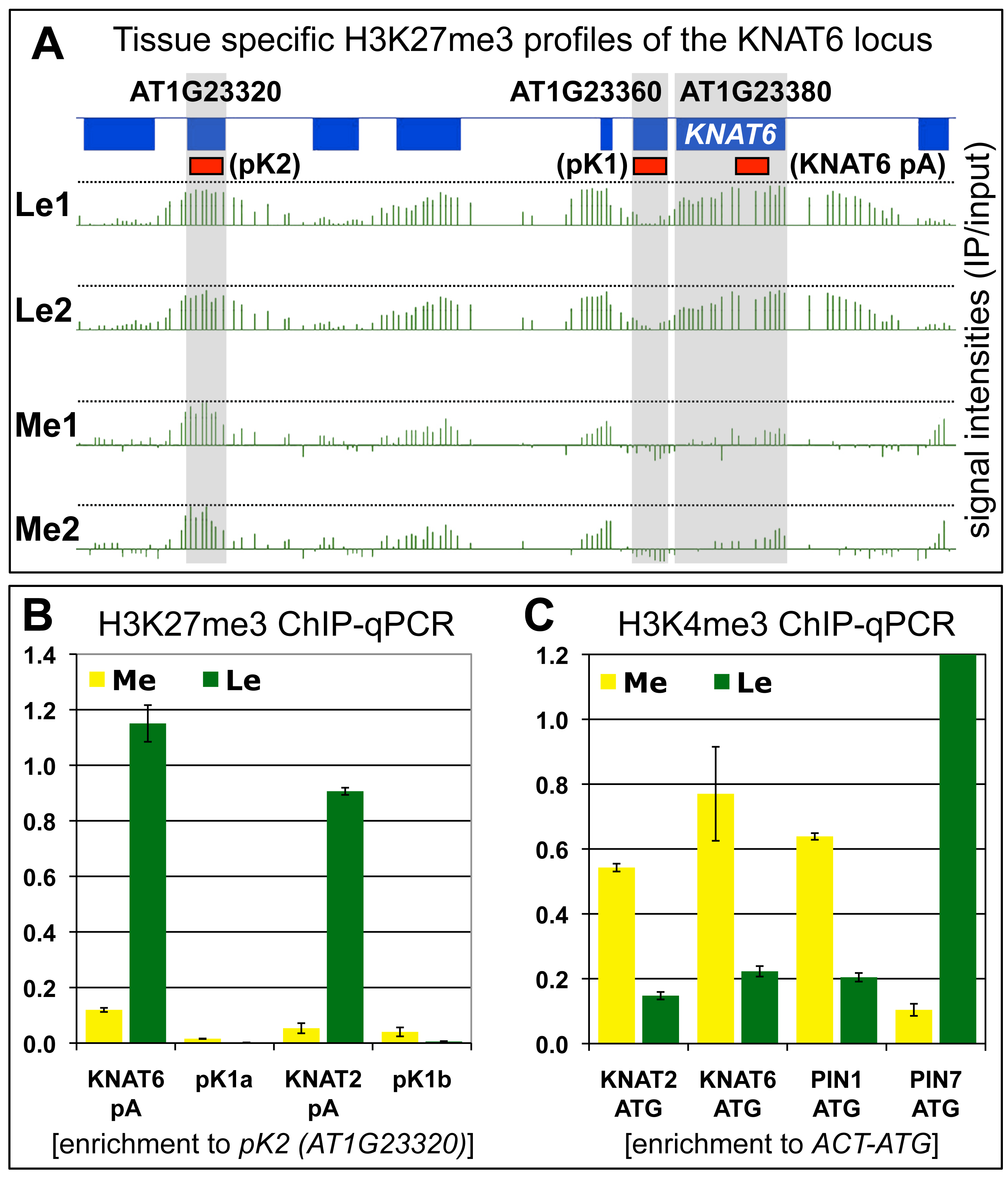

Supplement: Figure S1 — Tissue-specific differences of H3K4me3 and H3K27me3. (A) Comparison of the H3K27me3 profiles derived from meristem (Me) and leaf (Le) tissue reveal differences in the H3K27me3 abundance within the KNAT6 locus. Genes are depicted in blue, the signal intensity ratio (IP/input) of each array probe in green, the position of the qPCR-probes (KNAT6 pA, pK1, pK2) in red. (B, C) ChIP with H3K27me3 (B) and H3K4me3 (C) specific antibodies followed by qPCR analysis of the precipitates. Plant material was dissected from 9 weeks old, short day grown clv3 mutants. For the tissue comparison the data was normalized and is presented as enrichment to the corresponding reference loci “KNAT6 pK2” (AT1G23320) (for H3K27me3 ChIP) and “ACT pATG” (AT5G09810) (for H3K4me3 ChIP). Region pK1 (at1g23360) does not show enrichment in H3K27me3 in array (A) and ChIP-qPCR (B). (TIF) [file pgen.1002040.s001.tif]

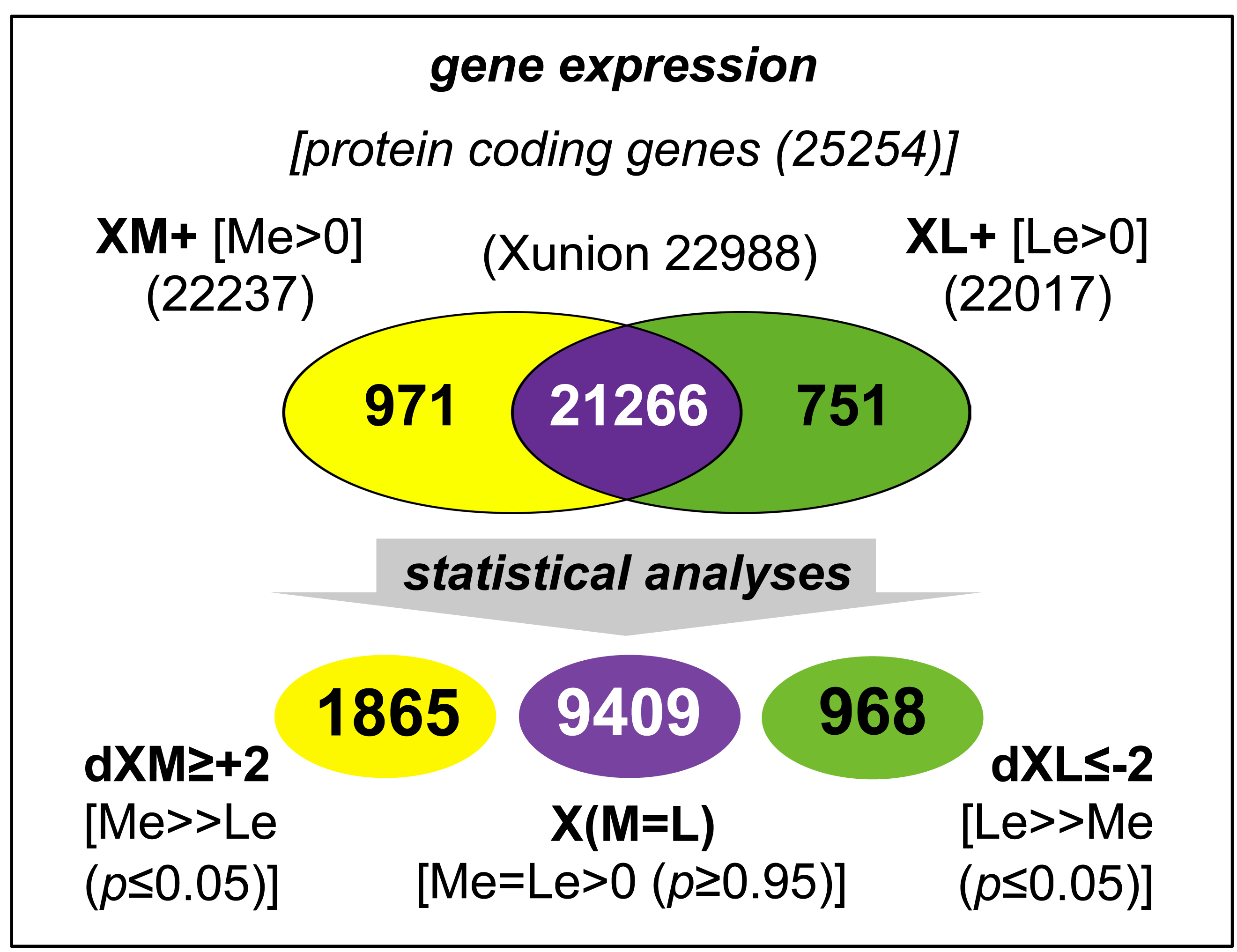

Supplement: Figure S2 — Identification of equally and differentially expressed genes. The diagram shows the number of expressed genes identified in meristem (Me) and leaf (Le) tissues as well as the intersection of both tissues (upper panel) (means of two biological replicates). Genes harbouring equal or differential expression levels were identified as described in methods (lower panel). (TIF) [file pgen.1002040.s002.tif]

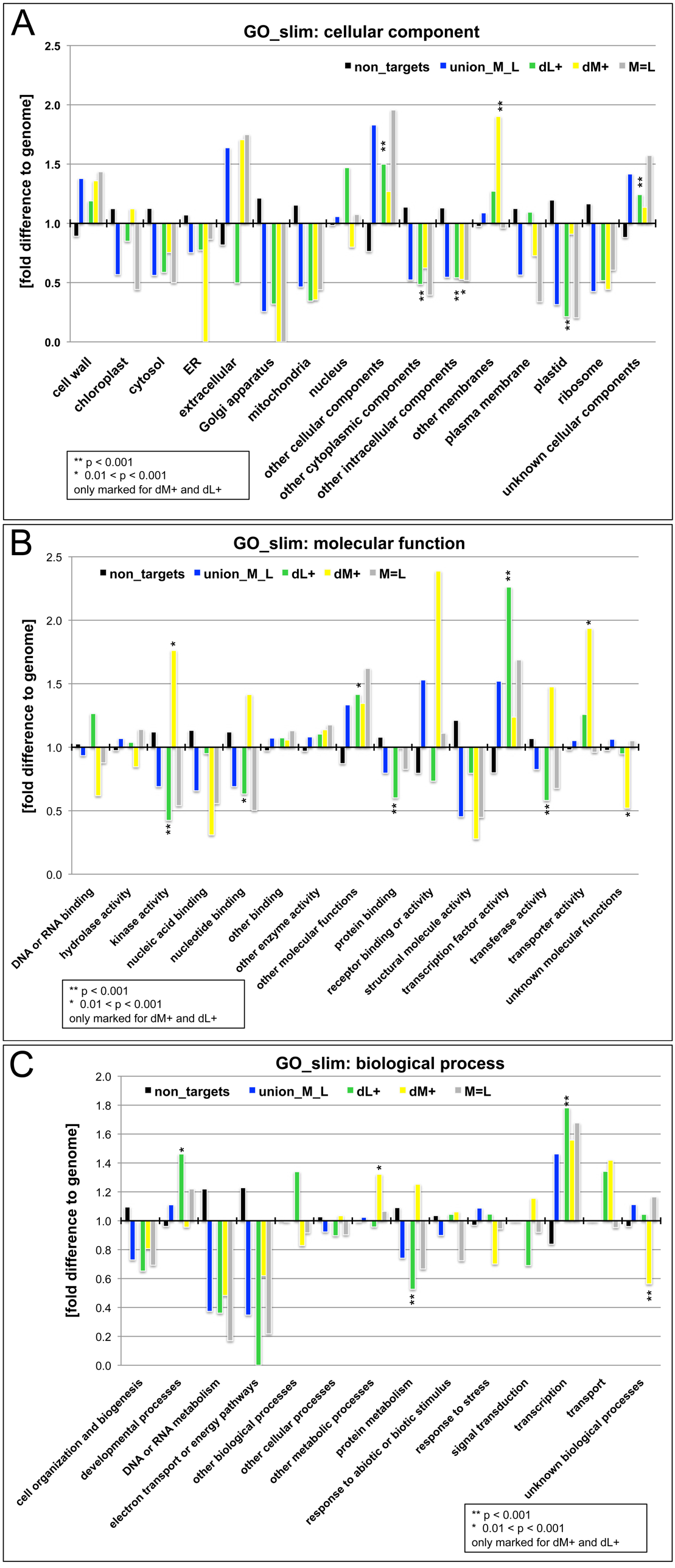

Supplement: Figure S3 — Functional categorization of H3K27me3 target genes by GO term analysis. (A–C) Frequency of H3K27me3 target (blue), non target (black), equally (grey), meristem- (yellow) and leaf-specifically (green) methylated genes within functional annotations (GO terms) relative to all protein coding genes of the TAIR8 Arabidopsis genome annotation. The analysis is split in the GO_slim analyses of cellular components (A), of molecular functions (B) and biological processes (C). Significant differences (hypergeometric test) are only given for dM+, dL+, Differences for target and non-target genes were all significant (p<0.001). (TIF) [file pgen.1002040.s003.tif]

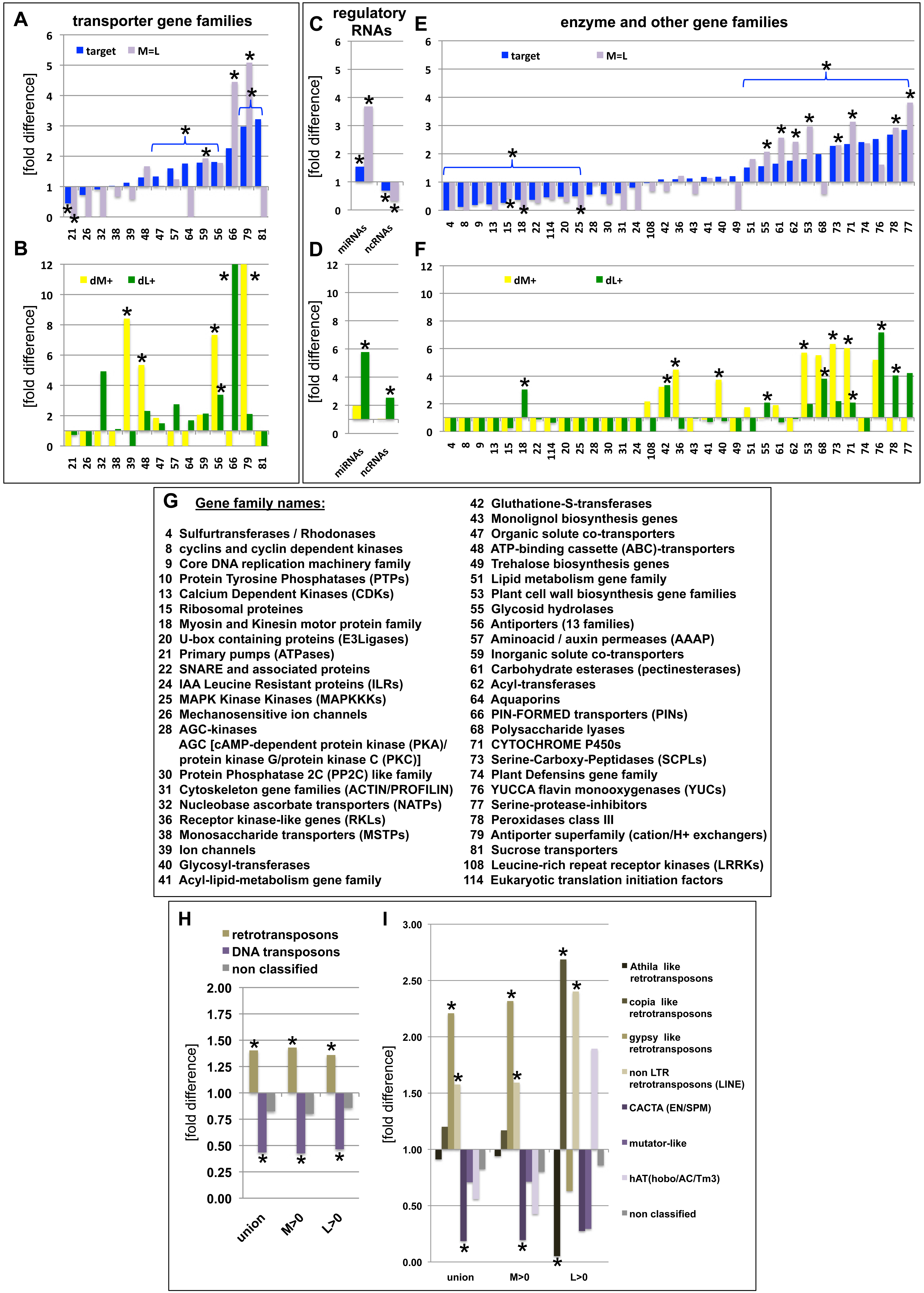

Supplement: Figure S4 — Abundance of H3K27me3 target genes in gene families. (A, C, E) Relative enrichment or depletion of H3K27me3 target genes (blue) or equally methylated genes (M = L, purple) in specific transporter (A), regulatory RNA (C), enzyme and other families (E). (B, D, F) Relative enrichment or depletion of meristem-specific (dM+, yellow) or leaf-specific (dM+, green) H3K27me3 target genes in specific transporter (B), small RNA (D), enzyme and other families (F). Y-axes indicate fold difference relative to the observed frequencies of specific subsets within the genome. Asterisks indicate significant differences (p≤0.05, χ2-test) compared to total number of genes for a specific subset (adjacent subsets with same significance are labelled with coloured brackets). Details of statistical analyses can be found in Table S3. Gene family names are shown in G. (H) Relative enrichment or depletion of H3K27me3 target genes in retrotransposon and DNA transposon genes. Retrotransposon and DNA transposon genes were grouped. (I) Relative enrichment or depletion of H3K27me3 target genes in subclasses of retrotransposon and DNA transposon genes (based on TAIR8 genome annotation). Y-axes indicate fold difference relative to the observed frequencies of specific subsets within the genome. Asterisks indicate significant differences (p≤0.05, χ2-test) compared to total number of genes for a specific subset. Details of statistical analyses can be found in Table S3. (TIF) [file pgen.1002040.s004.tif]

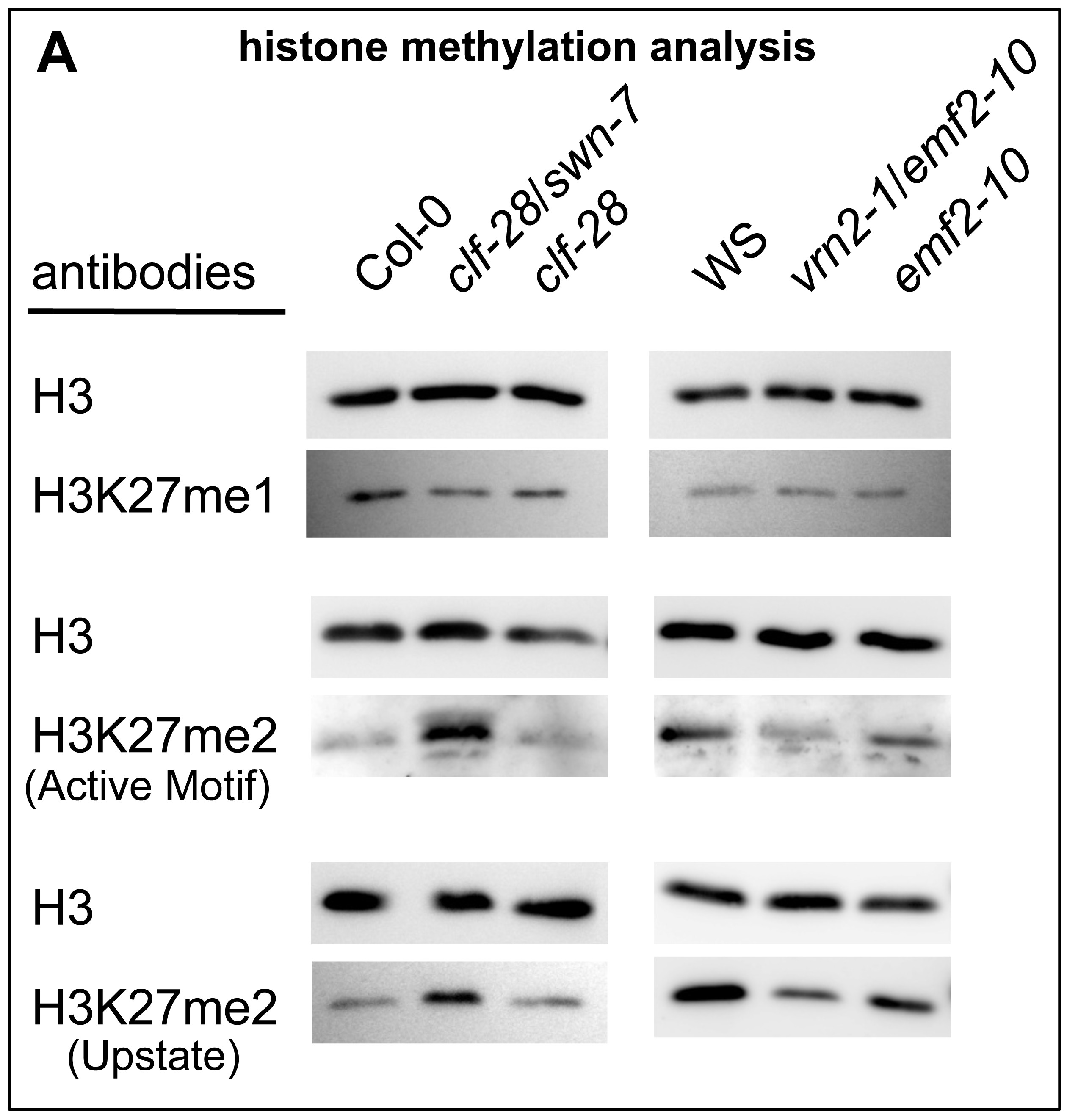

Supplement: Figure S5 — Immunoblot analyses of H3K27me1 and H3K27me2 in Pc-G mutants. Immunoblot analyses of H3K27me1 and H3K27me2 levels in wildtype and Pc-G mutants. Similar amounts of histone H3 was loaded as confirmed with a H3 specific antibody detecting the unmodified C-terminal part of H3. Same plant material was used as in Figure 6. (TIF) [file pgen.1002040.s005.tif]

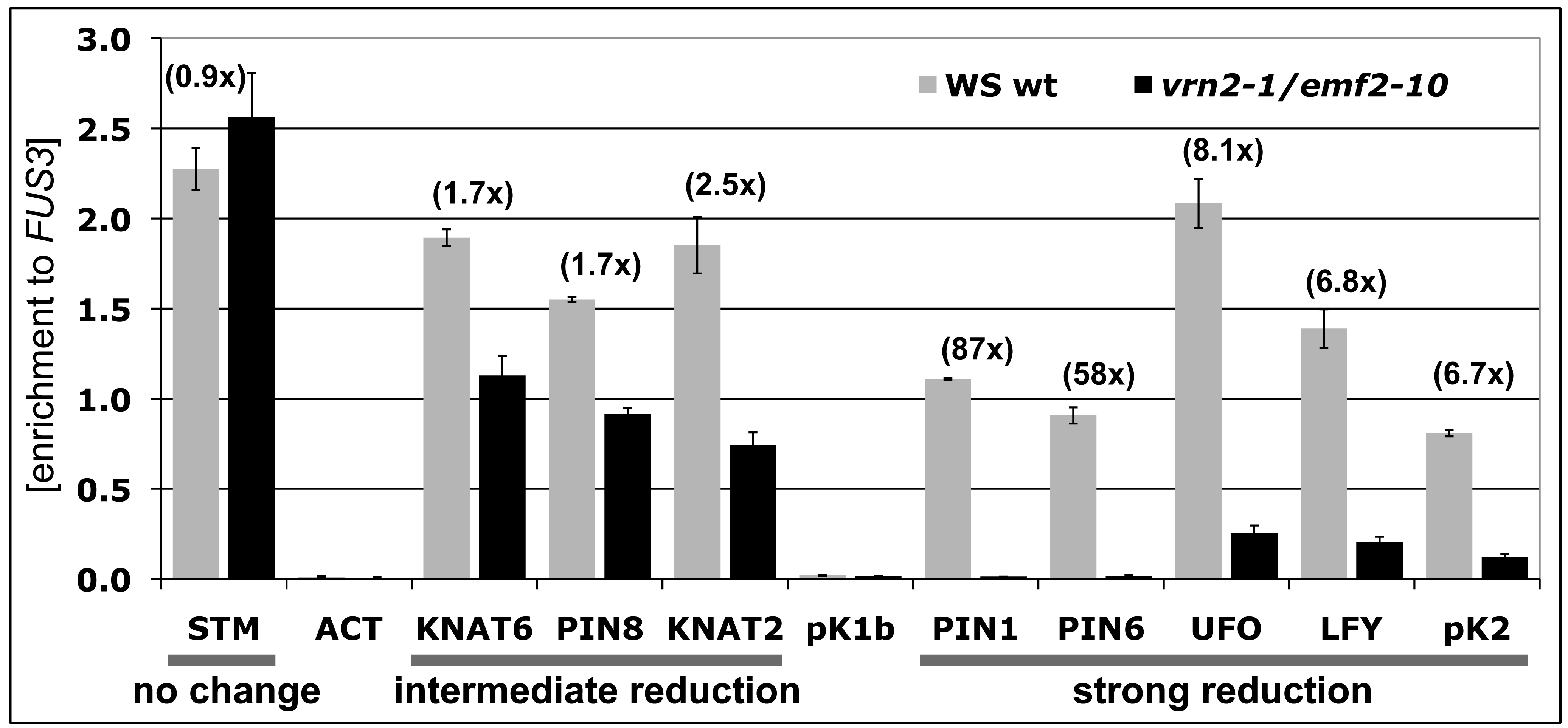

Supplement: Figure S6 — Gene-specific analyses of H3K27me3 in wildtype and vrn2-1/emf2-10 leaves. H3K27me3 target genes were analyzed in leaf material derived from 70d old, short day grown wild type and vrn2-1/emf2-10 plants by ChIP-qPCR. The qPCR data is presented as enrichment to the reference locus FUS3 (AT3G26790) as described in Figure 3, enrichment factors wt compared to vrn2-1/emf2-10 are indicated in brackets. The results are shown as mean values of two biological replicates. (TIF) [file pgen.1002040.s006.tif]

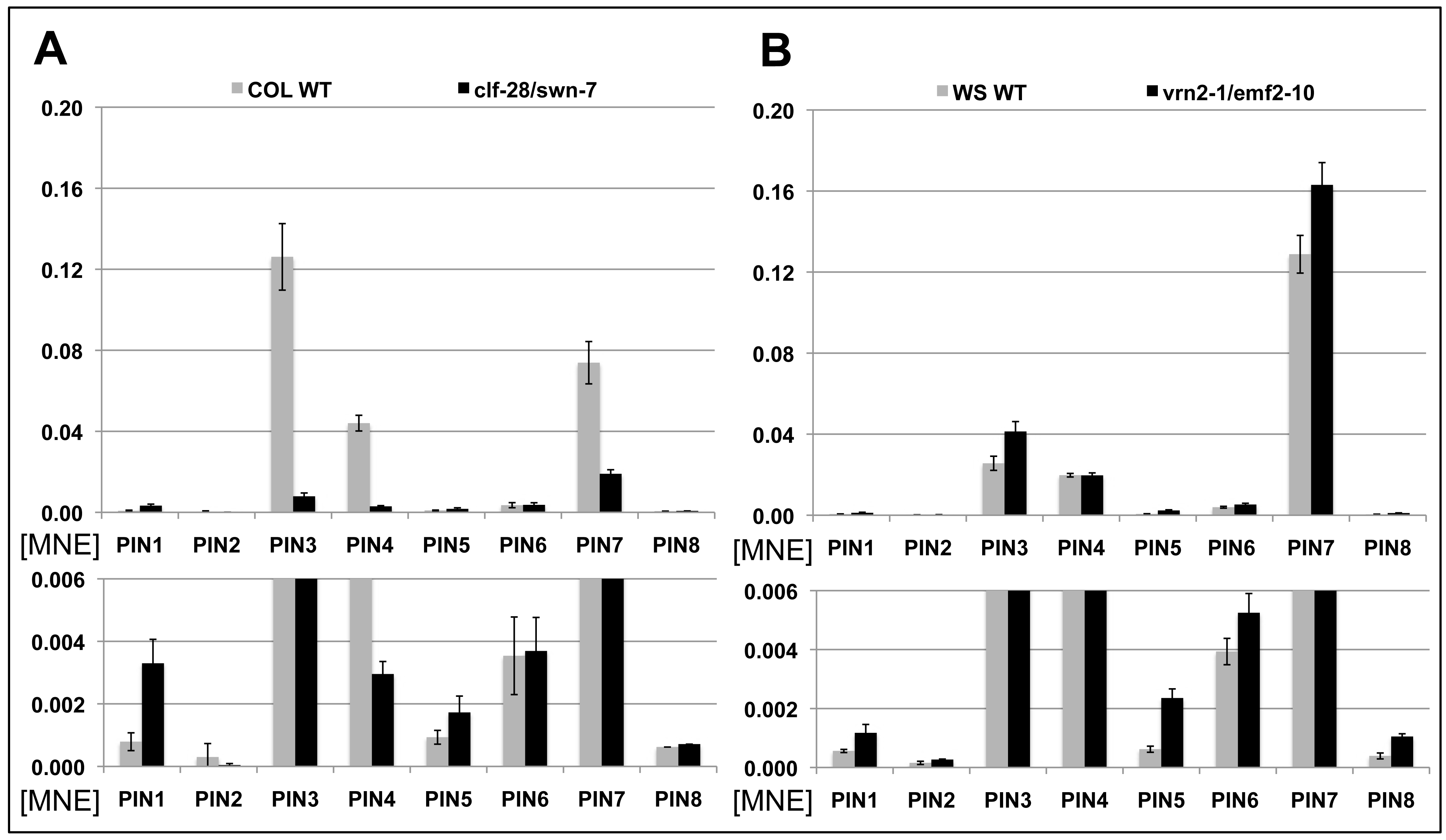

Supplement: Figure S7 — Regulation of PIN gene expression in Pc-G mutants. (A)–(B) qRT-PCR based expression analyses of (A) 9 d old wildtype and clf-28/swn-7 plants or (B) 45 days old leaves of wildtype and vrn2-1/emf2-10 plants. Expression values were normalized to At5g60390 (EF-1α). (TIF) [file pgen.1002040.s007.tif]

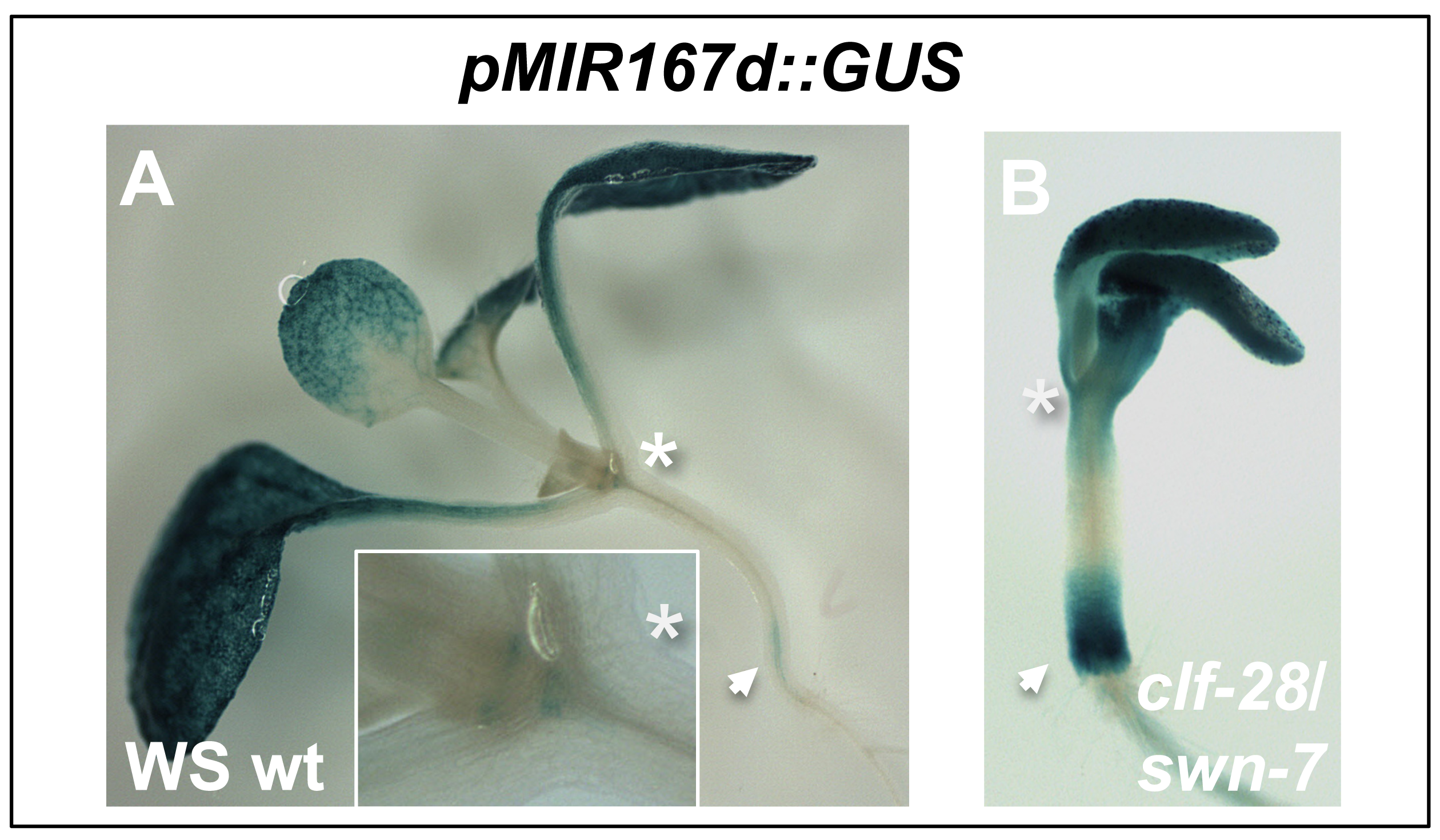

Supplement: Figure S8 — Colorimetric analyses of pMIR167d::GUS in wildtype and clf/swn mutants. Colorimetric analyses of pMir167d::GUS expression in 9 d old wildtype (A) and clf-28/swn-7 double mutants (F). Insets are close-ups. Asterisks mark meristematic region, white arrow base of hypocotyls. Scale bars indicate 1 cm. All plants were grown in short day conditions. (TIF) [file pgen.1002040.s008.tif]

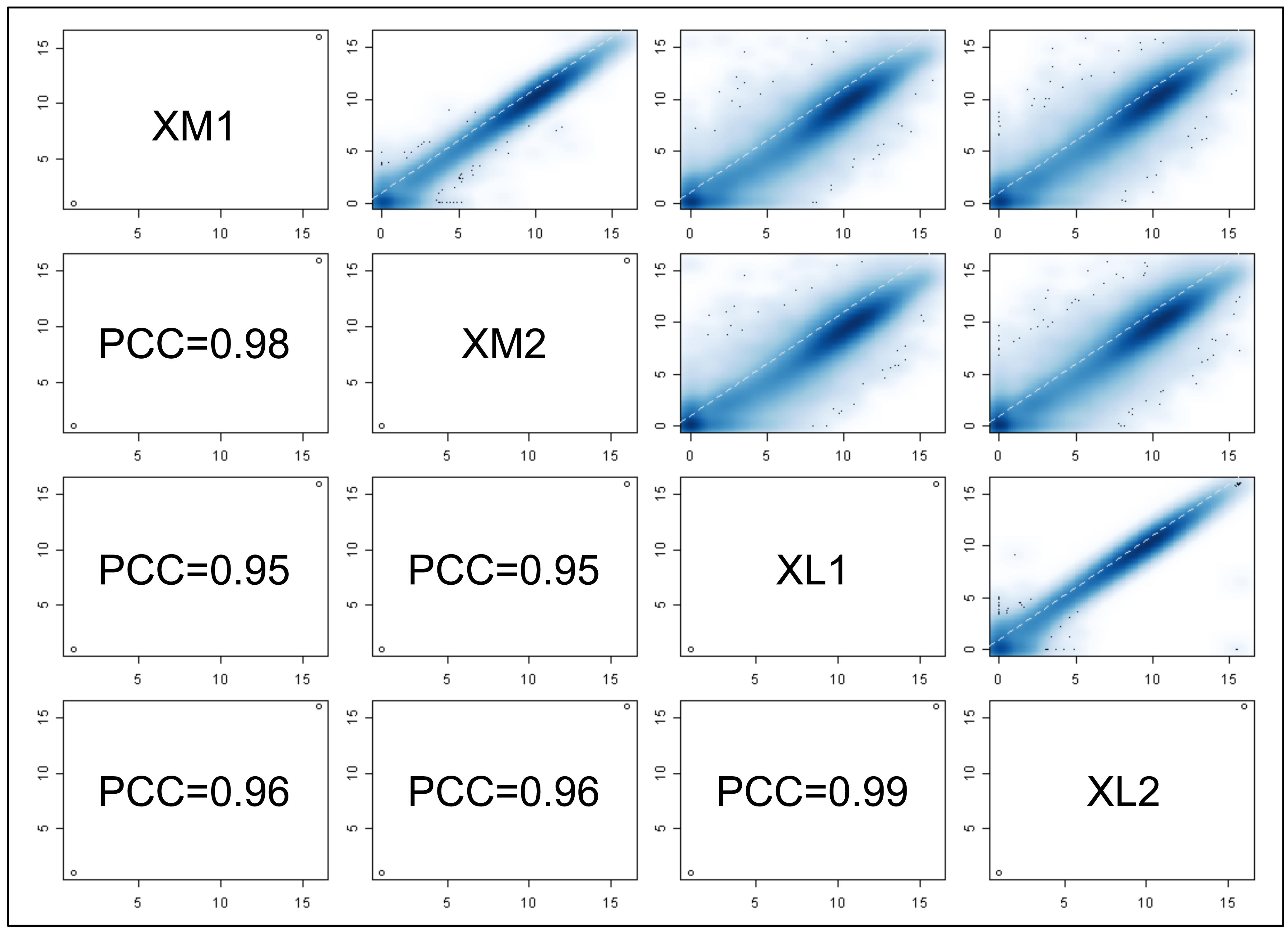

Supplement: Figure S9 — Comparison of expression arrays. Microarray hybridization intensities (XM1, XM2 = meristem expression profiles; XL1, XL2 = leaf expression profiles) were compared in a matrix of scatter plots revealing high overlap for biological replicates. The density of data points is represented by a darker colouration. White areas correspond to areas without data points. In low density areas single data points are indicated by single dots. The Pearson correlation coefficient (PCC) is shown below the diagonal. (TIF) [file pgen.1002040.s009.tif]

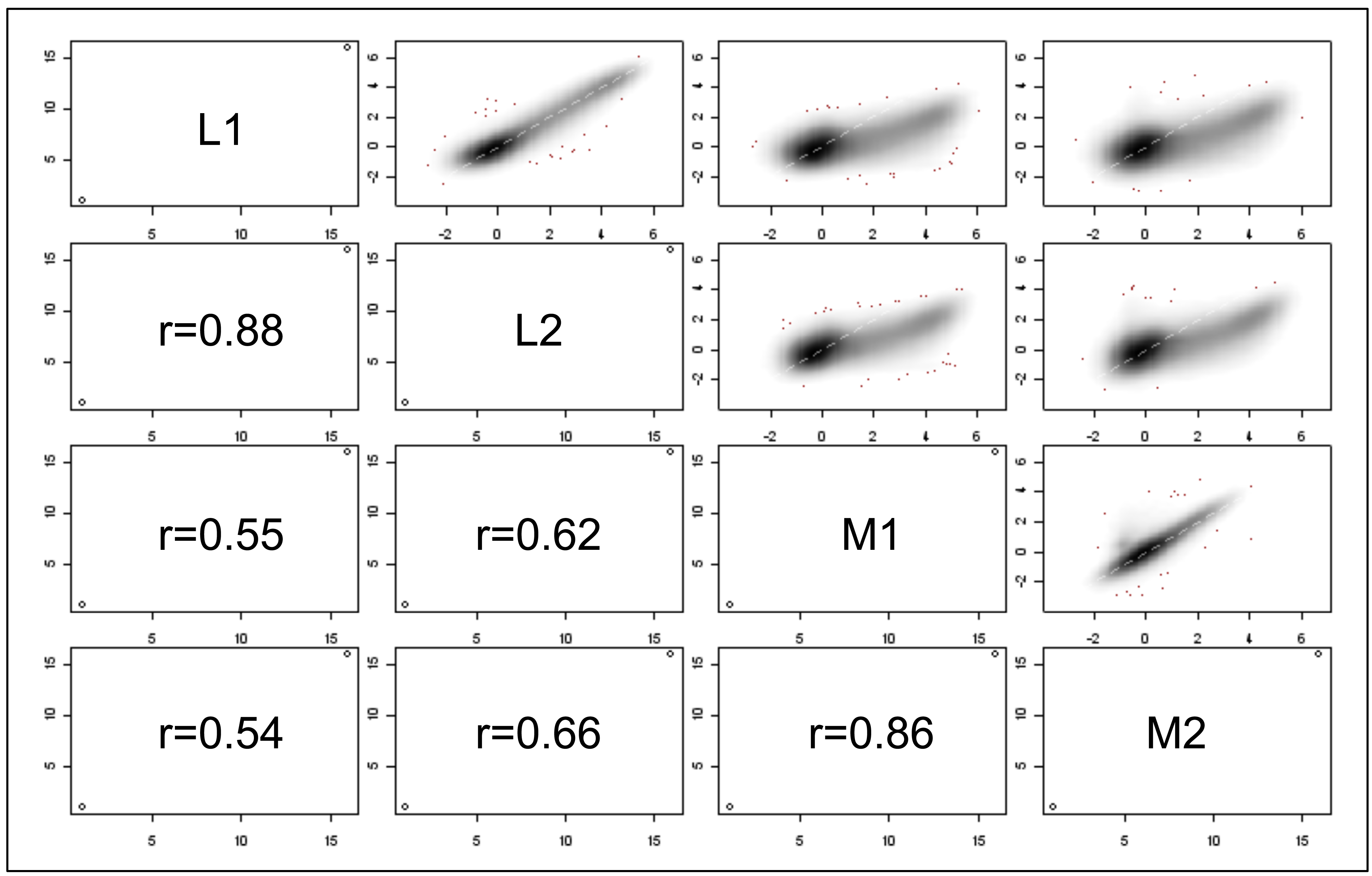

Supplement: Figure S10 — Correlation of H3K27me3 arrays (ChIP-chip) on the level of raw intensity values. The matrix plot correlates H3K27me3 ChIP-chip signal intensities between samples. L1 and L2 are biological replicates of leaf tissue, M1 and M2 of meristematic tissue. The upper triangle of the matrix displays scatter plots to compare each sample to the other samples. The axes represent the M-Values (M = log2(R)-log2(G) with R(red) = IP and G(green) = input) for all probes of the according sample. The intensity of shading of the scatter plot represents the density of data points within that area. Where density of data points is low single data points are shown. The lower triangle shows Spearman's rank correlation coefficients between different samples revealing high overlap between biological replicates. (TIF) [file pgen.1002040.s010.tif]

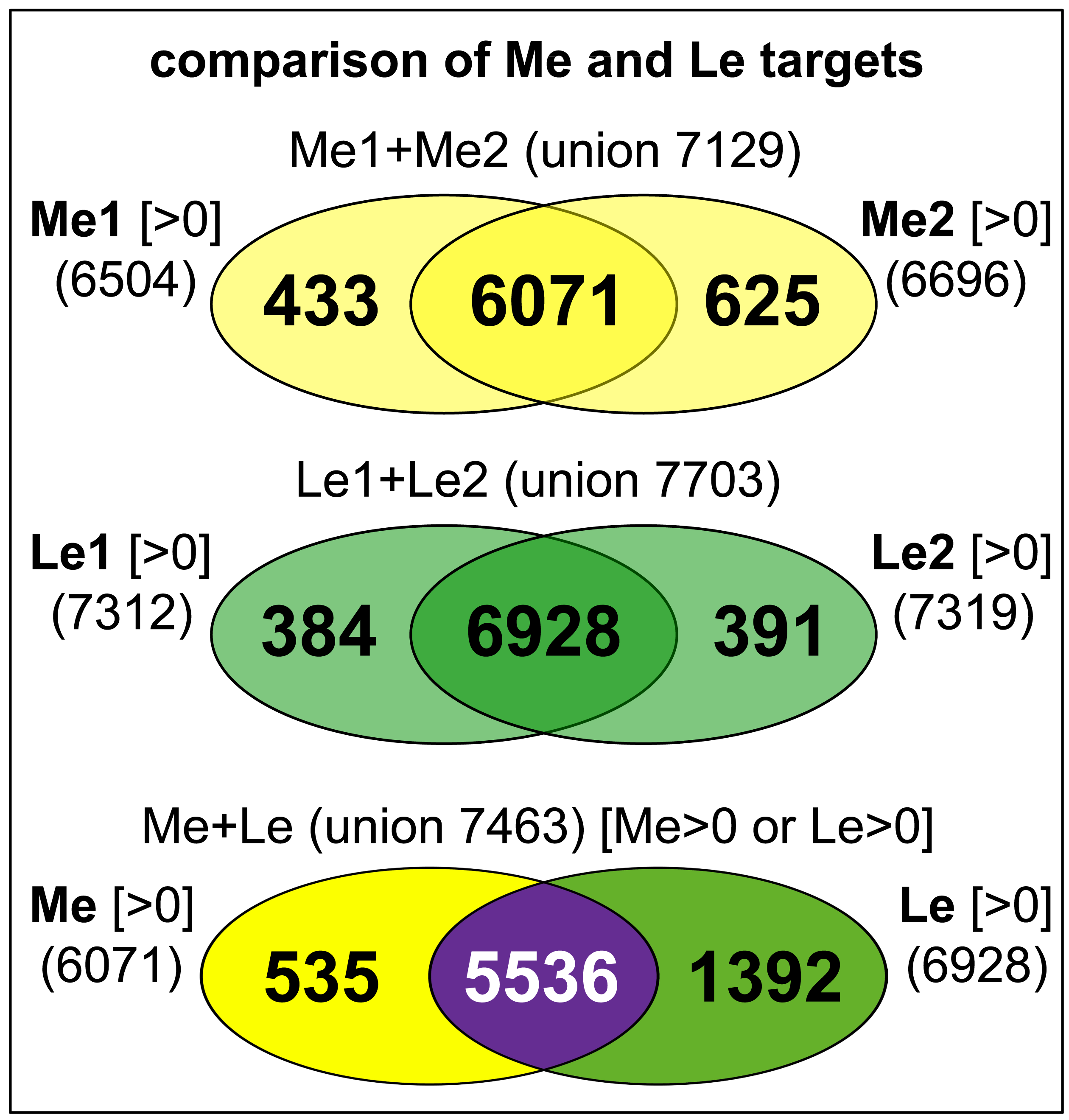

Supplement: Figure S11 — Comparison of identified H3K27me3 target genes in biological replicates. The diagram shows the identified numbers of H3K27me3 target genes for biological replicates for meristem (Me) or leaf (Le) material. The arrays of the same tissue share 85.2% (Me1 and Me2) and 89.9% (Le1 and Le2), respectively (two upper panels). Target genes identified in both replicates were used for the tissue comparison (lower panel and Figure 2). (TIF) [file pgen.1002040.s011.tif]

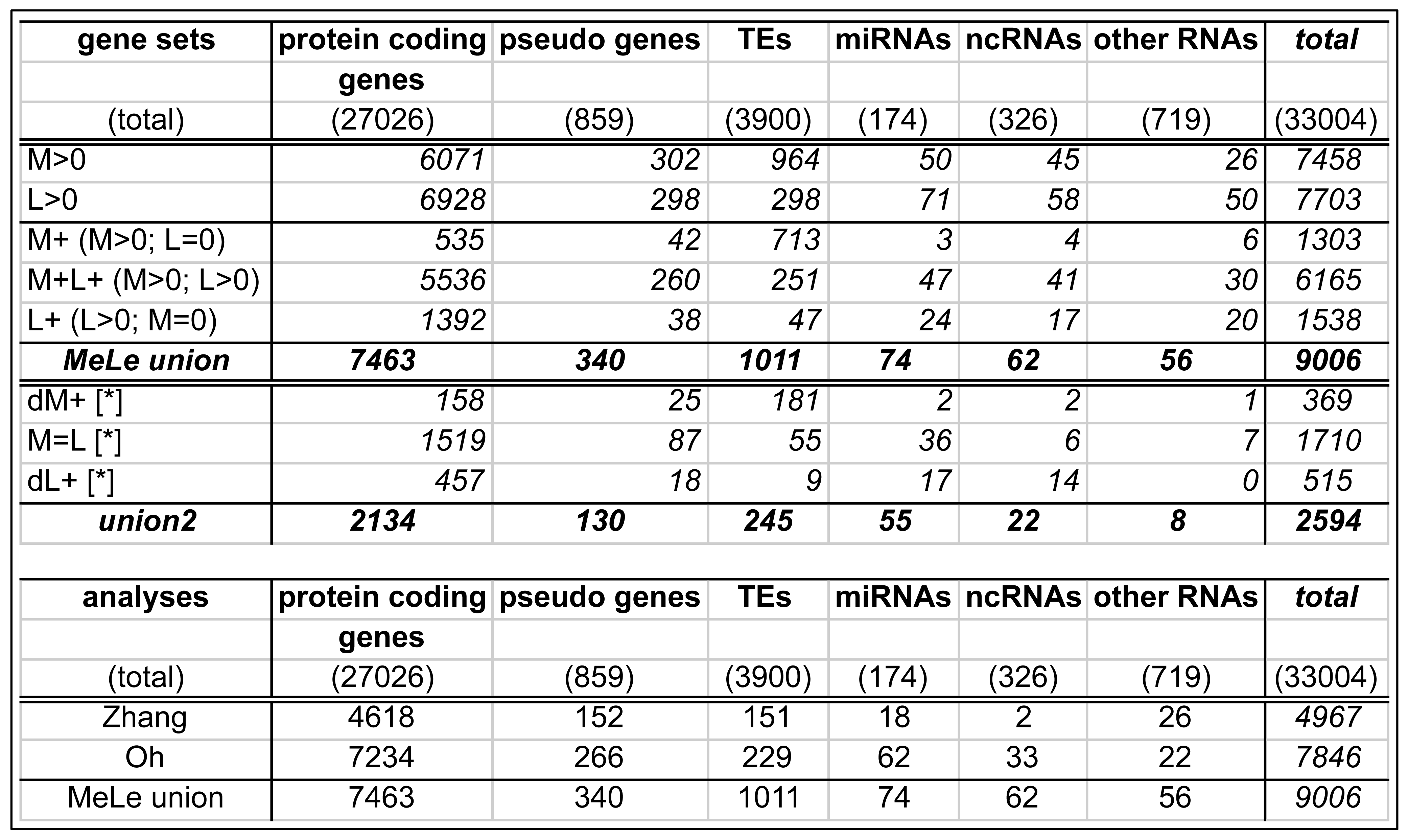

Supplement: Table S1 — Summary of all identified H3K27me3 target gene loci. All annotations of TAIR8 (www.arabidopsis.org) were analysed for their H3K27me3 coverage in leaf and meristematic tissue. The table shows the total numbers of identified target loci in comparison to the analysis of Zhang et al. [5] and Oh et al. [20]. TEs: transposable elements, ncRNAs: non coding RNAs. See methods for details on different subsets of meristem and leaf-specific H3K27me3 targets. (TIF) [file pgen.1002040.s012.tif]

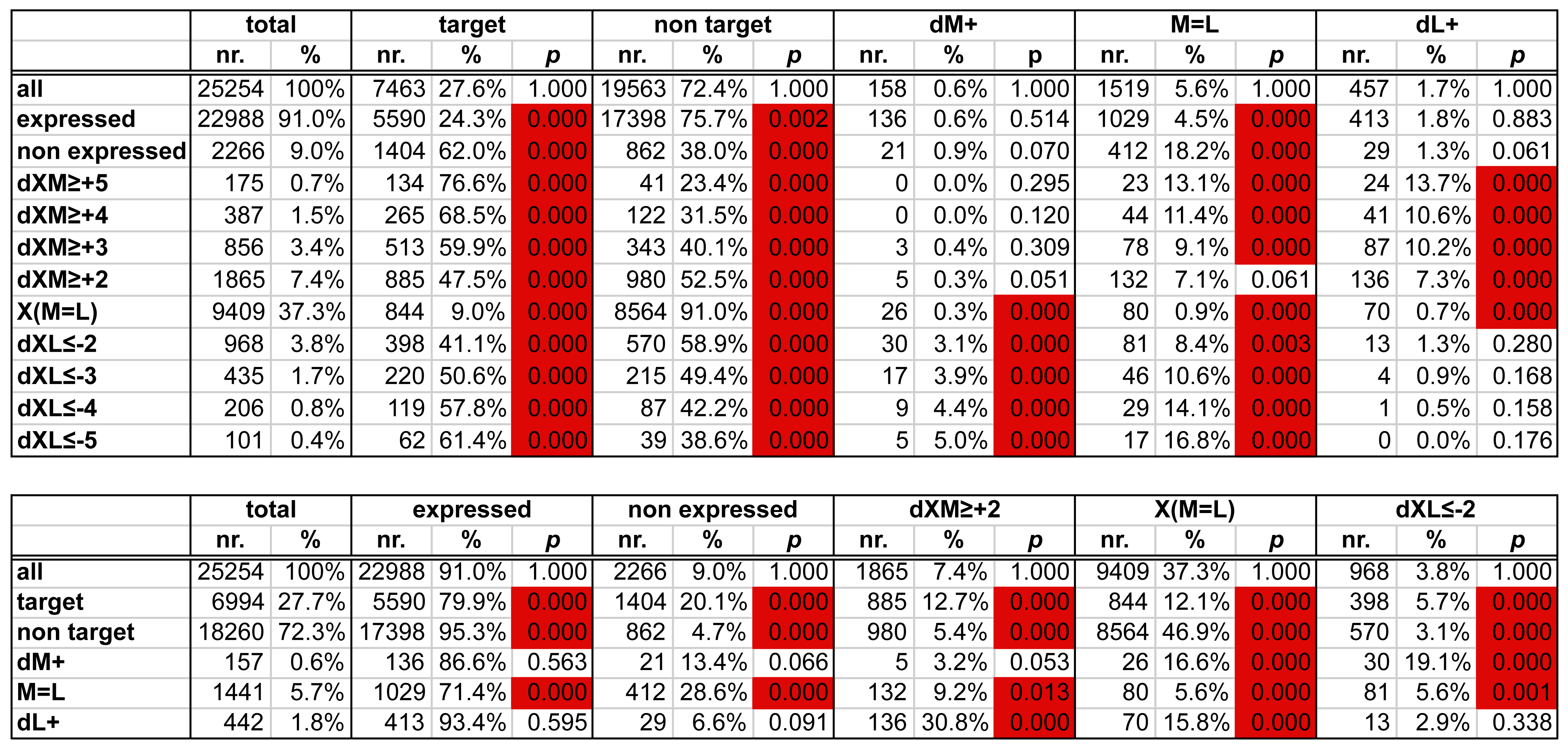

Supplement: Table S2 — Analysis of the significance of over- or under-representation of target genes within expression gene sets. Comparisons were performed between the frequency of a given H3K27me3 subset and the frequency of expression set in the genome (upper table) or between the frequency of a given expression set and the frequency of H3K27me3 subset in the genome (lower table). X2-test was applied to identify significantly different genes (p≤0.05). (TIF) [file pgen.1002040.s013.tif]

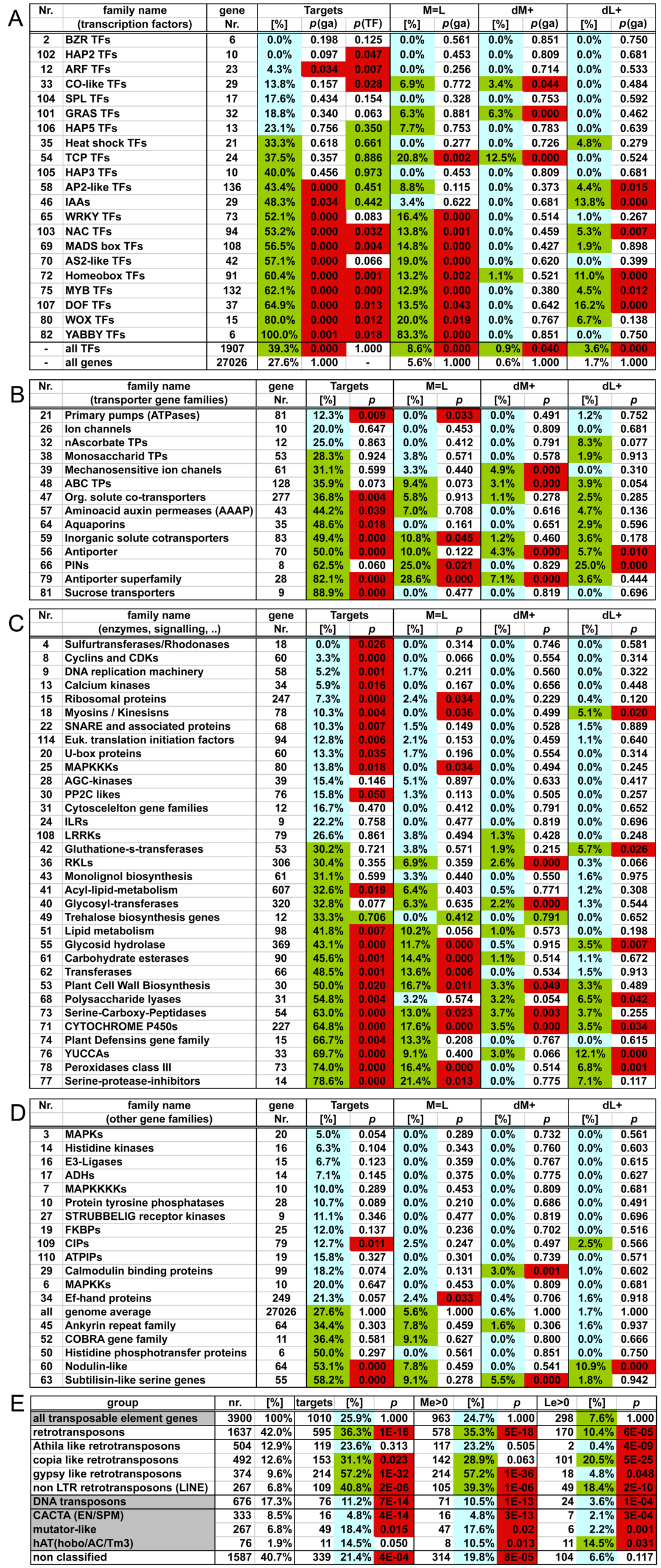

Supplement: Table S3 — Frequency of H3K27me3 target genes within gene families. The tables (A–E) show the percentages of H3K27me3 target genes, equally (M = L), meristem (dM+) and leaf (dL+) specifically methylated genes for transcription factor (A), transporter (B), other gene families (C, D) and transposable element genes (E). The probabilities that the observed frequencies within a gene family match the average representation in the genome [p(ga)] are given by a X2-test. Frequencies resulting in p≤0.05 were considered as significantly different. For transcription factor genes, the probabilities that the observed frequencies match the average of all transcription factor genes [p(TF)] were also calculated. (TIF) [file pgen.1002040.s014.tif]
